# Walking and Sun Protective Behaviors: Cross-Sectional Associations of Beneficial Health Factors

**DOI:** 10.3390/ijerph16132361

**Published:** 2019-07-03

**Authors:** Calvin P. Tribby, Frank M. Perna, David Berrigan

**Affiliations:** 1 Cancer Prevention Fellowship Program, Division of Cancer Prevention, National Cancer Institute, Bethesda, MD 20892, USA; 2 Health Behaviors Research Branch, Division of Cancer Control and Population Sciences, National Cancer Institute, Bethesda, MD 20892, USA

**Keywords:** sun protective behaviors, walking, sun safety, sunscreen, sun avoidance, sun protection

## Abstract

Sun protective behaviors and physical activity have the potential to reduce cancer risk. Walking is the most common type of physical activity in the United States, but it is unclear whether sun protective behaviors differ by categories of walking, such as leisure versus transportation walking. We examined whether sun protective behaviors varied by category or duration of walking in the 2015 National Health Interview Survey (N = 26,632), age ≥ 18 years. We used logistic regression to estimate sunscreen use, sun avoidance, and sun protective clothing use by four categories of walking (no reported walking, transportation only, leisure only, or walking for both) and separately for walking duration for the general population and sun-sensitive individuals. Prevalence of sunscreen use varied across walking categories and the odds of use were higher with longer walking duration for transportation and leisure compared to those who reported no walking. Sun avoidance varied across walking categories and the odds of avoidance were lower with longer duration leisure but not transportation walking. Sun protective clothing varied across walking categories and the odds of use were higher for longer duration transportation, but not leisure walking. Data on the concurrence of walking and sun protection is needed to further understand the relationship between these health behaviors. By examining leisure and transportation walking, we found variations in sun protective behaviors that may provide important insight into strategies to increase sun protection while promoting physical activity.

## 1. Introduction

The issue of co-occurring health behaviors is an ongoing focus in sun safety research [1]. Sun protective behaviors and physical activity are two behaviors that have the potential to reduce cancer risk. However, groups with higher levels of physical activity also have higher levels of sunburn and melanoma [2,3]. Walking is the most common type of physical activity in the United States, with 65% of adults reporting any walking in the past seven days [4]. Staying in the shade and sunscreen use are the most common types of sun protective behaviors, with 37% and 32%, respectively, reporting these behaviors when going outside on a warm sunny day [3]. It is unknown whether the prevalence of sun protective behaviors differs by categories of walking, such as leisure versus transportation walking. Walking typically occurs outside, on neighborhood streets, in parks, or on walking/jogging trails, although some occurs indoors, in shopping malls, or on a treadmill [5]. The distinction of walking category is important because potential misconceptions about using sun protection for only leisure-time activities may present unintentional risk for nonleisure exposure, such as walking for transportation [6].

Current research examining the association between physical activity and sun protective behaviors focuses on general levels of physical activity, such as total or leisure-time only, or specific contexts, such as athletics [7]. For example, college athletes are a high-risk group, yet over 50% reported never using sunscreen during sports [8]. Skateboarders are also a high-risk group, yet most do not apply sunscreen while skating [9]. The sun protective behaviors associated with the much more common physical activity of walking is not known. Furthermore, it is important to distinguish between types of walking, because walking trips taken for transportation purposes (to get someplace) have different behavioral and context characteristics than leisure trips (for fun, relaxation, or exercise). In general, walking for transportation accounts for about one-half of all walking trips [10]. Finally, those walking for transportation prefer more direct routes and are less sensitive to positive (e.g., a buffer between sidewalk and street or proximity to parks) and negative (e.g., safety from crime or safety from traffic) environmental features than leisure walkers [11,12].

Other factors associated with sun protective behaviors are classified as modifiable or nonmodifiable, and there are interactions between the two. Examples of modifiable factors are social, cognitive, and both family and individual knowledge, attitudes, and risk awareness [13]. Findings for cognitive factors, such as intention to use sun protection, include positive associations with worry about skin damage, self-efficacy for using sun protection, and perceived control over barriers to sun protection use [14]. Nonmodifiable factors include gender, age, socioeconomic status, ultraviolet radiation (UVR) of a geographic region, and personal and family history of melanoma [13]. There are gender differences in sun protective behaviors [15]. Women are more likely to use sunscreen overall, both on their face and other exposed skin, compared to men [16,17]. For other sun protective behaviors, women are more likely to seek shade, whereas men are more likely to wear long clothing to the ankle [3]. Finally, the relationship between self-reported sun protective behaviors and sunburn is not always direct: An analysis of the 2010 National Health Interview Survey (NHIS) found that the relationship between the two was insignificant [18]. However, a more recent analysis of the 2015 NHIS data that disaggregates sun protective behaviors finds that regular sunscreen users are more likely to experience sunburn compared to those who did not regularly use sunscreen [3].

The objective of this study is to examine the association between sun protective behaviors and walking for leisure or transportation after accounting for physical activity and other covariates.

## 2. Materials and Methods

### 2.1. Sample

We used the 2015 National Health Interview Survey (NHIS), Cancer Control Supplement conducted by the National Center for Health Statistics and administered by the United States Census Bureau. The NHIS is an annual household survey of a nationally representative sample of the U.S. noninstitutionalized adult (age ≥ 18 years) population with sample design and data collection details available online [19]. This study was exempt from review by the National Cancer Institute institutional review board because we used publicly available deidentified data.

The total sample of adults interviewed was 33,672 with an unconditional response frequency of 55.2% [19]. We excluded respondents who were unable to walk (N = 894) or had unknown or missing responses to sun protective behaviors or covariates (N = 6,146). Our analytic sample included 26,632 adults.

### 2.2. Sun Protective Behaviors

Sun protective behaviors were assessed with the question: “When you go outside on a warm sunny day for more than one hour, how often do you: Stay in the shade?; Wear a hat that shades your face, ears, and neck such as a hat with a wide brim all around?; Wear a long-sleeved shirt?; Wear long pants or other clothing that reaches your ankles?; and, Use sunscreen?” Following Holman et al., we created three behavior categories: Sunscreen use (with sun protective factor (SPF) ≥ 15), sun protective clothing (wide-brimmed hat, long-sleeved shirt, or long clothing to the ankle), and sun avoidance (staying in the shade or responding “do not go out in the sun” for any sun protective question) [3]. The responses for each sun protective behavior were “always,” “most of the time,” “sometimes,” “rarely,” and “never.” Participants were classified as performing each of the three summary behaviors if they reported “always” or “most of the time” to any of the behaviors in each category [3].

### 2.3. Walking Behaviors

The independent variable for transportation walking was based on response to the question: “During the past seven days, did you walk to get some place that took you at least 10 min?” The independent variable for leisure walking was based on response to the question: “Sometimes you may walk for fun, relaxation, exercise, or to walk the dog. During the past seven days, did you walk for at least 10 min for any of these reasons?” We used responses to these two questions to define four exclusive categories of walking: No reported walking (less than 10 min); walking for transportation only; walking for leisure only; or walking for both transportation and leisure. If respondents answered yes to either question, there were further questions about frequency and duration which provided estimates of weekly minutes of walking for transportation, leisure, or both [20].

### 2.4. Covariates

Covariates in the models were all self-reported from the NHIS and were used in previous research related to sun safety behaviors [3], walking [4], or sunburn [3], and included age, gender, education, ethnicity, marital status, U.S. or foreign born status, U.S. Census region, binge drinking status, smoking status, obesity or overweight, leisure-time physical activity level [21], health insurance status, those who repeatedly burn or freckle after two weeks in the sun, indoor tanning device use, self-applied sunless tanning product use, personal history of melanoma, family history of melanoma, ever had a skin exam, ever had cancer, and needs walking assistance. The ethnicity variable came from a re-coding of the NHIS questions on Hispanic origin (“Origin_I”) and race groups (“RaceRPI2”). The National Center for Health Statistics characterizes these two variables as race/ethnicity [19].

### 2.5. Statistical Analyses

We examined the associations between walking category and the three sun protective behaviors, adjusting for covariates. We used SAS (version 9.4; SAS Institute, Cary, NC, USA)-callable SUDAAN (version 11.0; Research Triangle Institute, Research Triangle Park, NC, USA) survey procedures to account for the complex sampling design, probability of selection, non-response, and sample weights. We used logistic regression models to estimate the prevalence of the three sun protective behaviors by walking category, adjusting for all covariates. Prevalence was estimated from predictive margins and the overall significance of variables used the adjusted Wald F-test. We stratified the models by an individual’s sun-sensitivity. Individuals were classified as sun-sensitive if they reported any skin burn to a question on what would happen to their skin after an hour in the sun without any protection [3].

To test differences in sun protective behaviors between walking categories, we used linear contrasts. These contrasts tested the difference of pairwise regression coefficients between the walking categories. We did not use multiple comparison adjustments to determine significance, however, we present results with 95% confidence intervals. Finally, we categorized the continuous walking duration variables among walkers (estimated minutes of weekly leisure or transportation walking) into quartiles (where the quartiles were restricted to those who walked) and present the adjusted odds ratios and 95% confidence intervals of sun protective behaviors for each quartile, with those who reported no walking as the reference category. We used *p* < 0.05 as the level of significance for all tests.

## 3. Results

### 3.1. Walking Prevalence

Those who reported regular sunscreen use were most likely to have reported walking for leisure (38.6%, (95% CI: 37.0–40.2%)), compared to the other three walking categories (Table 1). Those who reported regular sun avoidance behaviors were more likely to have reported no walking (39.6%, (95% CI: 38.3–40.8%)) than to have reported walking in either or both categories. Those who reported regular sun protective clothing use were more likely to have reported no walking (34.5%, (95% CI: 33.2–35.7%)) than to have reported walking in either or both categories. Finally, of those that reported at least one sunburn in the previous 12 months, 34.6% also reported walking for leisure only.

### 3.2. General Population

**Figure 1:**
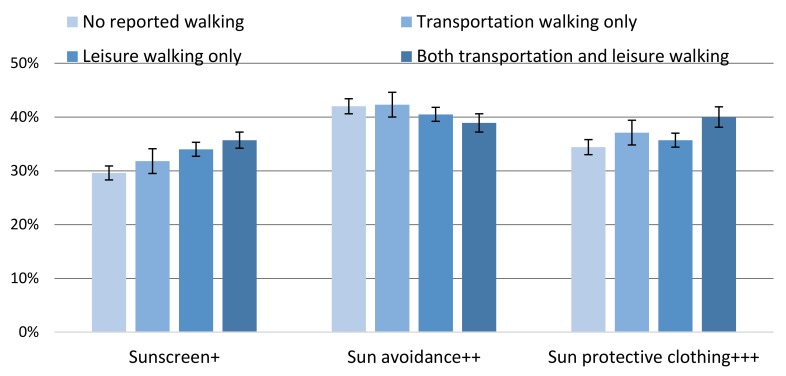
Weighted ^a^ and adjusted ^b^ prevalence (%) of U.S. adults age ≥ 18 years who reported regular sunscreen use ^c^, sun avoidance ^d^, and sun protective clothing ^e^ by walking category ^f^, National Health Interview Survey, 2015. (+ no reported walking vs. both, *p* < 0.0001; no reported walking vs. leisure, *p* < 0.0001; transportation vs. both, *p* < 0.01. ++ no reported walking vs. both, *p* < 0.01. +++ no reported walking vs. both, *p* < 0.0001; no reported walking vs. transportation, *p* < 0.05; transportation vs. both, *p* < 0.05; leisure vs. both, *p* < 0.01. ^a^ 95% confidence interval. ^b^ Covariates: Gender, age, ethnicity, education, marital status, foreign-born status, needs walking assistance, U.S. Census region, obesity or overweight, physical activity level, smoking status, binge drinking, tanning bed use, ever had a skin exam, ever had cancer, family history of melanoma, personal history of melanoma, sunless tanning product use, sunburn previous 12 months, skin reaction to sun after two weeks, and health insurance status. ^c^ Regular sunscreen use was defined as always or usually using sunscreen with SPF15+ (sun protection factor). ^d^ Response of always or usually staying in the shade or responding not going into the sun for any sun protective question. ^e^ Response of always or usually wearing long sleeve shirt, long clothing to the ankles, or a wide-brimmed hat on a warm sunny day. ^f^ Reported walking categories for at least 10 min in the past seven days.).

For the overall sample, the significant adjusted percentage point differences in sunscreen use between walking categories were: No reported walking vs. both (−6.1%, *p* < 0.0001); no reported walking vs. leisure (−4.4%, *p* < 0.0001); and transportation vs. both (−3.9%, *p* = 0.003) (Figure 1; full results in Supplementary Materials Table S1). The significant differences in sun avoidance behaviors between walking categories were: No reported walking vs. both (3.1%, *p* = 0.006) and transportation vs. both (3.4%, *p* = 0.02) (Figure 1; full results in Supplementary Materials Table S2). The significant differences in sun protective clothing use between walking categories were: No reported walking vs. both (−5.6%, *p* < 0.0001); no reported walking vs. transportation (−2.7%, *p* = 0.04); transportation vs. both (−2.9%, *p* = 0.04); and leisure vs. both (−4.3%, *p* = 0.0002) (Figure 1; full results in Supplementary Materials Table S3).

### 3.3. Sun-Sensitive Individuals

**Figure 2:**
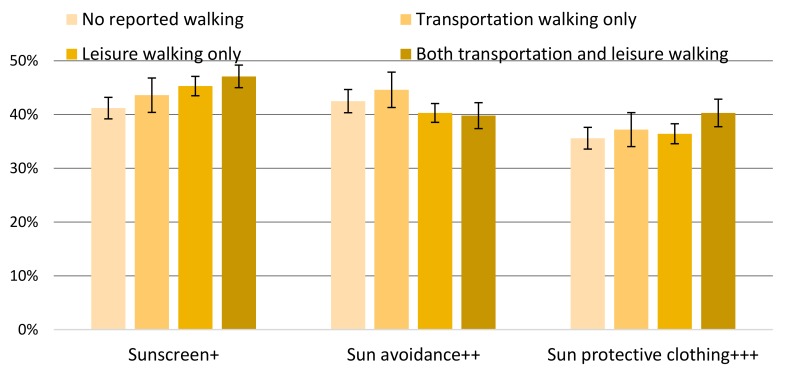
Weighted ^a^ and adjusted ^b^ prevalence (%) of sun-sensitive individuals ^c^ aged ≥ 18 years who reported regular sunscreen use ^d^, sun avoidance ^e^, and sun protective clothing ^f^ by walking category ^g^, National Health Interview Survey, 2015. (+ no reported walking vs. both, *p* < 0.0001; no reported walking vs. leisure, *p* = 0.0001. ++ transportation vs. both, *p* = 0.02; transportation vs. leisure, *p* = 0.02. +++ no reported walking vs. both, *p* = 0.007; leisure vs. both, *p* = 0.01. ^a^ 95% confidence interval. ^b^ Covariates: Gender, age, ethnicity, education, marital status, foreign-born status, needs walking assistance, U.S. Census region, obesity or overweight, physical activity level, smoking status, binge drinking, tanning bed use, ever had a skin exam, ever had cancer, family history of melanoma, personal history of melanoma, sunless tanning product use, sunburn previous 12 months, skin reaction to sun after two weeks, and health insurance status. ^c^ Sun-sensitivity defined as reporting any skin burn when not protected from the sun for 1 h. ^d^ Regular sunscreen use was defined as always or usually using sunscreen with SPF15+ (sun protection factor). ^e^ Response of always or usually staying in the shade or responding not going into the sun for any sun protective question. ^f^ Response of always or usually wearing long sleeve shirt, long clothing to the ankles, or a wide-brimmed hat on a warm sunny day. ^g^ Reported walking categories for at least 10 min in the past seven days.).

For sun-sensitive individuals, there were significant adjusted percentage point differences in sunscreen use between walking categories for: No reported walking vs. both (−7.0%, *p* < 0.0001) and no reported walking vs. leisure (−5.2%, *p* = 0.0001) (Figure 2; full results in Supplementary Materials Table S1). The significant differences in sun avoidance behaviors between walking categories were for: Transportation vs. both (4.8%, *p* = 0.02) and transportation vs. leisure (4.3%, *p* = 0.02) (Figure 2; full results in Supplementary Materials Table S2). The significant differences in sun protective clothing use between walking categories for: No reported walking vs. both (−4.7%, *p* = 0.007) and leisure vs. both (−4.0%, *p* = 0.01) (Figure 2; full results in Supplementary Materials Table S3).

### 3.4. Walking Duration

**Figure 3:**
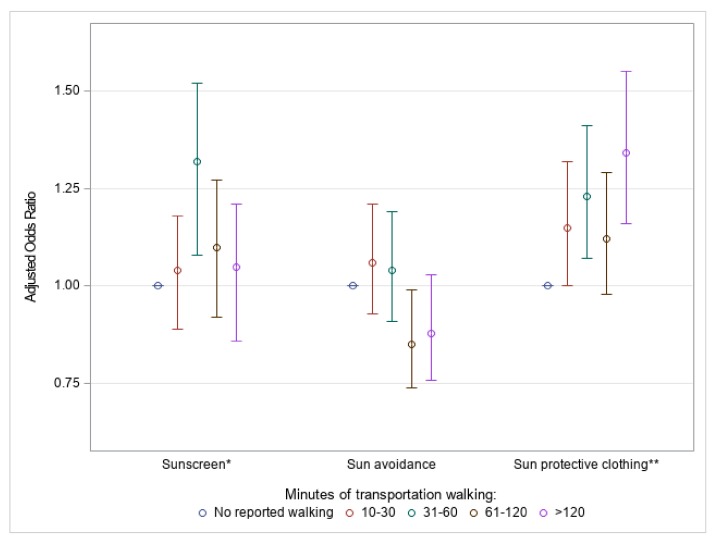
Weighted ^a^ adjusted ^b^ odds ratios for sun protective behaviors ^c^ by quartile of weekly transportation walking minutes for U.S. adults aged ≥ 18 years, National Health Interview Survey, 2015. (* *p* = 0.02; ** *p* = 0.0002. ^a^ 95% confidence interval; reference: No reported walking (<10 min of weekly transportation walking). ^b^ Covariates: Gender, age, ethnicity, education, marital status, foreign-born status, needs walking assistance, U.S. Census region, obesity or overweight, physical activity level, smoking status, binge drinking, tanning bed use, ever had a skin exam, ever had cancer, family history of melanoma, personal history of melanoma, sunless tanning product use, sunburn previous 12 months, skin reaction to sun after two weeks, and health insurance status. ^c^ Response of always or usually using SPF 15+ on a warm sunny day (sunscreen); always or usually staying in the shade or responding not going into the sun for any sun protective question (sun avoidance); always or usually wearing long sleeve shirt, long clothing to the ankles, or a wide-brimmed hat on a warm sunny day (sun protective clothing)).

The duration of transportation walking for the general population had a significant (*p* = 0.02) association with adjusted odds of sunscreen use, with the category of 31–60 min having the largest adjusted odds ratio, compared to those who reported no walking: 1.32 (95% CI: 1.12–1.56) (Figure 3). The duration of transportation walking was not significant (*p* = 0.05) for sun avoidance behaviors but had a significant (*p* = 0.0002) association with sun protective clothing (Figure 3). Those who reported more than 120 min per week of transportation walking had 34% higher odds of sun protective clothing use compared to those who reported no walking (adjusted OR, 1.3 (95% CI: 1.1–1.6)). Full results, including sun-sensitive individuals, in Supplementary Materials Tables S4–S6.

**Figure 4:**
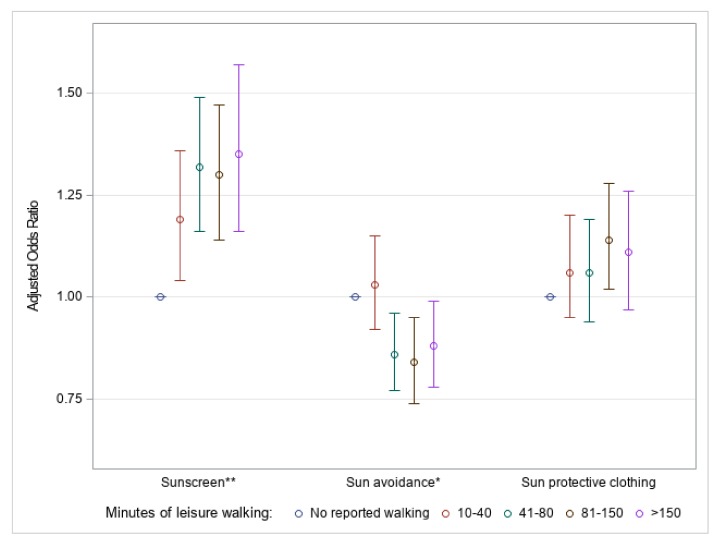
Weighted ^a^ adjusted ^b^ odds ratios for sun protective behaviors ^c^ by quartile of weekly leisure walking minutes for U.S. adults aged ≥ 18 years, National Health Interview Survey, 2015. (** *p* < 0.0001; * *p* = 0.0008. ^a^ 95% confidence interval; reference: No reported walking (<10 min of weekly leisure walking). ^b^ Covariates: Gender, age, ethnicity, education, marital status, foreign-born status, needs walking assistance, U.S. Census region, obesity or overweight, physical activity level, smoking status, binge drinking, tanning bed use, ever had a skin exam, ever had cancer, family history of melanoma, personal history of melanoma, sunless tanning product use, sunburn previous 12 months, skin reaction to sun after two weeks, and health insurance status. ^c^ Response of always or usually using SPF 15+ on a warm sunny day (sunscreen); always or usually staying in the shade or responding not going into the sun for any sun protective question (sun avoidance); always or usually wearing long sleeve shirt, long clothing to the ankles, or a wide-brimmed hat on a warm sunny day (sun protective clothing)).

The duration of leisure walking for the general population had a significant (*p* < 0.0001) association with sunscreen use. Those who walked more than 150 min had the greatest adjusted odds of sunscreen use compared to those who reported no walking: 1.35 (95% CI: 1.16–1.57) (Figure 4). The duration of leisure walking had a significant (*p* = 0.0008) negative association with odds of sun avoidance behaviors, compared to those who reported no walking (Figure 4). Those who walked more than 150 min per week for leisure had 16% (95% CI: 6–26%) lower odds of sun avoidance behaviors compared to those who reported no walking. Duration of leisure walking did not have a significant (*p* = 0.17) association with odds of sun protective clothing (Figure 4). Full results, including sun-sensitive individuals, can be found in Supplementary Materials Tables S4–S6.

## 4. Discussion

Walking is a common type of physical activity accessible to most people [4]. The associations between physical activity and sun protective behaviors are mixed [3,7]. This research found that category or duration of walking was associated with differences in prevalence of sun protective behaviors. More research is needed into the behavior context of outdoor activities and sun protection behaviors to improve sun safety and promote physical activity [1].

Associations between physical activity and sun protective behaviors depend on the type of physical activity. For example, we found the prevalence of regular sunscreen use was consistently higher for those who walked for both transportation and leisure compared to those who reported no walking, while controlling for leisure-time physical activity. This increased prevalence of sunscreen use with higher walking activity is consistent with other research findings on higher sunscreen use with higher total leisure-time physical activity [3,22]. Yet, if the type of physical activity is organized sports, the prevalence of sunscreen use is lower [7,8,23]. Sun avoidance behaviors, such as seeking shade or not going outdoors, is negatively related with leisure-time activity, such as in resorts or at beaches [24,25]. Similarly, we found that longer duration leisure walking was negatively associated with sun avoidance. Finally, leisure-time physical activity is generally negatively associated with sun protective clothing use [24,26]. We did not find a similar association between leisure-time walking and sun protective clothing. However, for transportation only and both transportation and leisure walking we found higher sun protective clothing use. This is a novel finding that may be related to the behaviors and context of transportation walking and requires further research to explore.

Guidelines for sun protection behaviors in the U.S. are, when possible, to avoid prolonged exposure to the sun, especially between 10 a.m. to 4 p.m., wear tightly weaved or high-SPF clothing, wear a wide-brimmed hat and sunglasses, and seek shade [27]. For sunscreen use, it is advised to use in combination with other protective practices, select a product with at least SPF 15+, apply liberally, reapply every 2 h, and reapply immediately after swimming and heavy perspiration [27]. The effectiveness of these sun protective behaviors is contingent on specific conditions and appropriate use [16,28]. This has led to inconsistent associations between sun protective behaviors and sunburn [29,30]. One issue with sun protective behaviors is the unintended consequence of individuals using certain behaviors to extend duration of sun exposure. For example, research has found that sunscreen use is associated with increased sunburn rates, because individuals believe it can increase the amount of time for safe exposure [31,32,33]. The relationship between sun protective clothing and sunburn is inconclusive, likely due variations in material and dependence on use of other sun protective strategies [3]. Sun avoidance behaviors, such as seeking shade or not going outside, are associated with fewer sunburns [3,28,34]. Depending on UVR conditions and individuals’ sun sensitivity, sunburn may occur in as little as 10 min, so even for relatively short walking trips, protection is advised [29]. More research is needed on which sun protective behaviors are suitable and more likely to be adopted by individuals for transportation and leisure walking trips. Additional research may explore whether those who walk for longer duration may have higher general health awareness and whether this explains some of the higher prevalence of sun protection in these groups. In general, knowledge of physical activity guidelines was higher for those who met the guidelines [35]. However, whether this knowledge crosses over to other health behavior domains needs further scrutiny.

The measurement and operationalization of sun protective behaviors in research usually combines separate behaviors into a single additive index. This assumes that a higher score, indicating regularly performing more behaviors, provides better protection from sunburn [36,37,38,39]. However, recent research suggests that an index underperforms in predicting sunburn, when compared to a decision tree which assessed patterns of behaviors [40]. This is likely because the inclusion of sunscreen use, which is associated with increased sunburns, reduced the predictive utility of an index. Therefore, we chose not to combine all sun behaviors together into an index. Additionally, our hypothesis was that sun protective behaviors may vary by walking behavior, which would not be evident with a sun protective behavior index.

A challenge for sun protective behavior interventions is to substantially increase use among individuals in a population [30,41]. A further challenge is to maintain lasting behavior change [42]. A different approach may be multicomponent community-wide interventions, which combine individual-level intervention with community or policy supports [43] or modifications of environments to facilitate sun protection. A review of environmental and policy interventions to promote sun protective behaviors in recreation and tourism settings have mixed results [44] and the provision of shade at vacation destinations may not be utilized by vacationers [25]. Some modifications of the built environment, such as bus stop shelters, store front canopies, or street trees, may provide some sun protection for transportation walking [45]. Further, designing public spaces and buildings with shade provision may increase sun protection for transportation and leisure walking [46]. Future research may assess the effectiveness of shade provision for transportation and leisure walking trips, where sun avoidance may not be feasible.

### Limitations

The research goal of this study was to examine the association between walking and sun protective behaviors while controlling for overall physical activity. However, our measure of physical activity was a limitation with these data. First, it included only leisure-time physical activity. Second, when leisure-time physical activity was excluded, overall, the findings were attenuated compared to models which controlled for leisure-time physical activity (Supplementary Materials Tables S1–S6). This attenuation may be due to controlling for the effect of overall physical activity. However, it may also be due to measurement issues. There was potential overlap between reported walking and leisure-time activity, so inclusion of this variable could be over-controlling for the associations of interest. For example, some respondents could have reported walking during the walking questions and may have also considered and reported leisure and transportation walking as part of their leisure-time activity. It is likely that the true association may lie somewhere between the models’ estimates for each sun protective behavior, and our main findings adjusting for physical activity may be conservative.

Other limitations of this research were temporal coincidence, temporal duration, seasonality, and walking location. First, temporal coincidences of behavior based on NHIS questions were unclear. Questions about sun protective behaviors were asked separately from walking trip questions. Therefore, we cannot determine coincidence of sun protective behaviors while walking from these data. Second, the temporal duration of the sun protective questions (warm sunny day for at least an hour) did not match the walking questions (at least 10 min). Therefore, it was problematic to infer whether sun protective behavior performed while outside for at least an hour was relevant to walking trips of shorter duration. A third limitation was that the season of survey administration may influence responses to walking questions, which asked about the previous seven days. Respondents may have given different responses to walking questions whether it asked in the warmer months versus the colder months. However, the sun protective behavior questions asked about a warm sunny day in general, making the comparison between walking behaviors and sun protective behaviors lead to potentially different responses if asked in summer versus winter. Lastly, the location of the walking trips was not specified. For example, leisure walking may have taken place indoors, such as a shopping mall, and therefore sun protective behaviors were irrelevant [47].

Some common limitations of research on self-reported behaviors apply to this study. Specifically, self-reported physical activity, such as walking, may over- or under-estimate directly measured physical activity, depending on intensity of episodes and individuals’ gender [48]. Self-reported sun protective behaviors also may over- or under-estimate directly observed use. For example, survey measures of sunscreen use are not likely prone to recall error or social desirability bias [49]. Studies of sun protective clothing use indicate the potential for social desirability bias, as a higher percentage of adults reported clothing use than was observed [50], while other research found no systematic over- or under-reported use than was observed [51]. Finally, this study uses cross-sectional data and cannot infer causality between sun protective behaviors and walking.

Over-adjustment may bias the causal effects between exposure and outcome toward null or cause spurious associations, depending on the relationship of the intermediate variable [52]. Unnecessary adjustment may not necessarily bias the effect between exposure and outcome, but may influence the precision [52]. Both of these potential outcomes are not necessarily detrimental to this study if they exist, primarily because we are not investigating causality. The main aim of this study is to determine whether the phenotypes of people, defined by category or duration of walking, is associated with sun protective behaviors. We are not proposing to know the direction of the relationship between walking and sun protective behaviors. The measures of both these behaviors are imprecise: Walking is any reported walking (more than 10 min) in the past seven days; the sun protective behaviors are regular use on a warm, sunny day.

Although over-adjustment may bias the association toward the null, which may affect some of our results, such as the adjusted association between regular sun avoidance and duration of transportation walking, there are many other limitations with these data that are more likely to explain the lack of association. As mentioned above, the questions do not ask about sun protective clothing use on walking trips. Therefore, while we may postulate that there is a causal effect of duration of transportation walking on sun avoidance behaviors, the survey questions do not allow us to formally test this association, including the testing of potential mediators and moderators. This is because the questions are worded as such that we cannot infer temporality of behaviors. Instead, we use the covariates to normalize the different attributes between the study populations of interest: Those in different walking categories or durations. This may seem like an unnecessary adjustment, however, to compare our results with previous analyses with these data, it is best to use the same set of covariates. Finally, some covariates are not significantly associated with the outcome in the bivariate analyses but are in the adjusted analyses and vice versa. We leave all the covariates in the adjusted analyses, so the reader may assess these changes, rather than omitting covariates and their estimates. We provide unadjusted estimates in the Supplementary Materials Tables for the readers’ reference for bivariate associations between sun protective behaviors and all covariates.

## 5. Conclusions

We found evidence of differences in the prevalence of sun protective behaviors by walking category and duration. Research is needed on public health messaging to encourage physical activity and effective sun protection for transportation and leisure walking trips. Finally, for walking trips where sun avoidance may not be feasible, such as walking for transportation, an investment in built environment features to provide shade may reduce sunburn risk. However, little is known about the cost effectiveness of such strategies, and research is needed to determine their potential public health benefit.

## Figures and Tables

**Table 1:** Weighted unadjusted prevalence (%) and 95% confidence intervals in exclusive walking categories ^a^ (rows sum to 100%) for U.S. adults by selected characteristics, National Health Interview Survey, 2015.

	N	No Reported Walking	Transportation Walking Only	Leisure Walking Only	Both Transportation and Leisure Walking
Total	26,632	36.0 (35.1–36.9)	11.1 (10.5–11.6)	32.2 (31.3–33.1)	20.8 (20.1–21.5)
Regular sunscreen use (SPF15+) ^b^					
Yes	8322	26.2 (24.9–27.6)	10.0 (9.1–10.9)	38.6 (37.0–40.2)	25.2 (23.7–26.6)
No	18,310	40.7 (39.6–41.8)	11.6 (10.9–12.3)	29.0 (28.0–30.0)	18.7 (17.9–19.4)
Regular sun avoidance ^c^					
Yes	11,154	39.6 (38.3–40.8)	11.3 (10.5–12.2)	30.7 (29.4–31.9)	18.4 (17.4–19.4)
No	15,478	33.5 (32.3–34.6)	10.9 (10.2–11.6)	33.2 (32.1–34.3)	22.4 (21.4–23.5)
Regular sun protective clothing use ^d^					
Yes	10,443	34.5 (33.2–35.7)	11.3 (10.4–12.2)	31.7 (30.4–32.9)	22.6 (21.4–23.8)
No	16,189	36.8 (35.7–38.0)	10.9 (10.2–11.6)	32.5 (31.3–33.6)	19.8 (18.9–20.7)
At least 1 sunburn in past 12 months					
Yes	8878	30.6 (29.2–31.9)	11.2 (10.3–12.2)	34.6 (33.2–36.0)	23.6 (22.2–24.9)
No	17,754	39.0 (37.9–40.1)	11.0 (10.3–11.6)	30.8 (29.8–31.8)	19.2 (18.4–20.1)
Gender					
Women	14,324	35.0 (34.0–36.1)	9.9 (9.2–10.6)	35.6 (34.4–36.7)	19.5 (18.5–20.5)
Men	12,308	36.9 (35.7–38.2)	12.3 (11.4–13.1)	28.7 (27.5–29.9)	22.1 (21.1–22.2)
Age group					
18–24	2508	35.1 (32.3–37.8)	15.6 (13.6–17.6)	25.6 (23.1–28.1)	23.8 (21.3–26.2)
25–34	4872	33.1 (31.3–34.9)	11.4 (10.2–12.7)	32.7 (30.8–34.6)	22.7 (21.1–24.3)
35–44	4414	34.4 (32.3–36.5)	11.0 (9.9–12.0)	32.7 (30.6–34.7)	22.0 (20.2–23.8)
45–64	9083	35.3 (33.9–36.5)	10.6 (9.7–11.5)	33.8 (32.2–35.4)	20.2 (18.9–21.5)
≥65	5755	43.0 (41.2–44.8)	8.0 (7.0–9.0)	32.8 (31.1–34.4)	16.3 (14.9–17.6)
Ethnicity					
White, non-Hispanic	16,709	35.0 (33.9–36.2)	9.9 (9.2–10.6)	34.5 (33.4–35.7)	20.6 (19.6–21.5)
Black, non-Hispanic	3235	43.0 (40.6–45.3)	13.7 (11.9–15.5)	23.1 (21.1–25.1)	20.3 (18.2–22.4)
Hispanic	4467	38.0 (36.1–39.9)	13.0 (11.6–14.4)	28.7 (26.9–30.4)	20.6 (18.8–21.9)
Other ethnicity	2221	29.9 (27.2–32.6)	13.2 (11.4–15.0)	32.4 (29.7–35.1)	24.4 (21.9–26.9)
Skin reaction after 2 weeks in sun					
Very dark tan	3335	36.1 (33.8–38.4)	12.3 (10.7–13.8)	30.8 (28.5–33.1)	20.8 (18.9–22.6)
Moderate tan	8376	34.2 (32.8–35.6)	10.5 (9.6–11.5)	34.0 (32.7–35.4)	21.3 (20.0–22.5)
Mild tan	8298	34.9 (33.5–36.4)	11.6 (10.6–12.6)	31.6 (30.2–32.9)	21.9 (20.7–23.1)
Burn repeatedly or freckle	4509	32.9 (30.9–34.9)	10.6 (9.3–11.8)	36.0 (33.8–38.2)	20.5 (18.7–22.2)
Do not go in the sun	2114	55.6 (52.4–58.8)	10.2 (8.5–11.8)	19.7 (17.3–22.1)	14.6 (12.6–16.7)
Physical activity level ^e^					
Inactive	7932	63.5 (62.0–65.1)	12.5 (11.4–13.5)	15.2 (14.1–16.2)	8.9 (8.0–9.7)
Insufficiently active	5387	33.7 (32.0–35.5)	11.2 (10.0–12.4)	37.4 (35.6–39.2)	17.7 (16.3–19.1)
Sufficiently active	4355	22.4 (20.7–24.2)	11.8 (10.5–13.2)	41.8 (39.8–43.9)	23.9 (22.0–25.7)
Highly active	8958	21.5 (20.2–22.7)	9.5 (8.7–10.3)	38.2 (36.7–39.7)	30.8 (29.3–32.3)

^a^ Reported walking categories for at least 10 min in the past seven days. ^b^ Regular sunscreen use was defined as always or usually using sunscreen with SPF15+ (sun protection factor). ^c^ Sun avoidance was defined as always or usually staying in the shade or responding not going into the sun for any sun protective question. ^d^ Sun protective clothing was defined as always or usually wearing at least one: Wide-brimmed hat, long sleeved shirt, or long clothing to the ankles. ^e^ Individuals were categorized into four activity levels based on the 2008 Physical Activity Guidelines for Americans: Highly active (>300 min/week of light or moderate-intensity aerobic activity, 150 min of vigorous-intensity aerobic activity, or an equivalent combination per week (i.e., moderate-intensity equivalent activity)), sufficiently active (150–300 min/week of moderate-intensity equivalent activity), insufficiently active (some activity, but less than 150 min/week of moderate-intensity equivalent activity), and inactive (no light-to-moderate or vigorous-intensity aerobic activity for at least 10 min).

## Supplementary Materials

The following are available online at https://www.mdpi.com/1660-4601/16/13/2361/s1, Table S1: Weighted unadjusted and adjusted proportion of U.S. adults and sun-sensitive individuals ^a^ aged ≥ 18 years who reported regular sunscreen use ^b^ by exclusive walking category ^c^ and covariates, National Health Interview Survey, 2015, Table S2: Weighted unadjusted and adjusted proportion of U.S. adults and sun-sensitive individuals ^a^ aged ≥ 18 years who reported regular sun avoidance ^b^ by exclusive walking category ^c^ and covariates, National Health Interview Survey, 2015, Table S3: Weighted and adjusted proportion of U.S. adults and sun-sensitive individuals ^a^ aged ≥ 18 years who reported regular sun protective clothing use ^b^ by exclusive walking category ^c^ and covariates, National Health Interview Survey, 2015, Table S4: Weighted and adjusted odds ratios for U.S. adults and sun-sensitive individuals ^a^ aged ≥ 18 years for regular sunscreen use ^b^ by quartile of weekly transportation and leisure walking minutes ^c^ and covariates, National Health Interview Survey, 2015, Table S5: Weighted and adjusted odds ratios for U.S. adults and sun-sensitive individuals ^a^ aged ≥ 18 years for regular sun avoidance ^b^ by quartile of weekly transportation and leisure walking minutes ^c^ and covariates, National Health Interview Survey, 2015, Table S6: Weighted and adjusted odds ratios for U.S. adults and sun-sensitive individuals ^a^ aged ≥ 18 years for regular sun protective clothing use ^b^ by quartile of weekly transportation and leisure walking minutes ^c^ and covariates, National Health Interview Survey, 2015.

## References

[1] Geller A.C., Jablonski N.G., Pagoto S.L., Hay J.L., Hillhouse J., Buller D.B., Kenney W.L., Robinson J.K., Weller R.B., Moreno M.A. (2018). Interdisciplinary Perspectives on Sun Safety. JAMA Dermatol..

[2] Moore S.C., Lee I.-M., Weiderpass E., Campbell P.T., Sampson J.N., Kitahara C.M., Keadle S.K., Arem H., de Gonzalez A.B., Hartge P. (2016). Association of Leisure-Time Physical Activity with Risk of 26 Types of Cancer in 1.44 Million Adults. JAMA Intern. Med..

[3] Holman D.M., Ding H., Guy G.P., Watson M., Hartman A.M., Perna F.M. (2018). Prevalence of Sun Protection Use and Sunburn and Association of Demographic and Behaviorial Characteristics with Sunburn Among US Adults. JAMA Dermatol..

[4] Ussery E.N., Carlson S.A., Whitfield G.P., Watson K.B., Berrigan D., Fulton J.E. (2017). Walking for Transportation or Leisure Among, U.S. Women and Men—National Health Interview Survey, 2005–2015. MMWR Morb. Mortal. Wkly. Rep..

[5] Eyler A.A., Brownson R.C., Bacak S.J., Housemann R.A. (2003). The Epidemiology of Walking for Physical Activity in the United States. Med. Sci. Sports Exerc..

[6] Dixon H.G., Hill D.J., Karoly D.J., Jolley D.J., Aden S.M. (2007). Solar UV Forecasts: A Randomized Trial Assessing Their Impact on Adults’ Sun-Protection Behavior. Health Educ. Behav..

[7] Jinna S., Adams B.B. (2013). Ultraviolet Radiation and the Athlete: Risk, Sun Safety, and Barriers to Implementation of Protective Strategies. Sports Med..

[8] Wysong A., Gladstone H., Kim D., Lingala B., Copeland J., Tang J.Y. (2012). Sunscreen use in NCAA collegiate athletes: Identifying targets for intervention and barriers to use. Prev. Med..

[9] Fernández-Morano T., de Troya-Martín M., Rivas-Ruiz F., Fernández-Peñas P., Padilla-España L., Sánchez-Blázquez N., Buendía-Eisman A. (2017). Sun Exposure Habits and Sun Protection Practices of Skaters. J. Cancer Educ..

[10] Santos A. (2011). Summary of Travel Trends: 2009 National Household Travel Survey.

[11] Brownson R.C., Hoehner C.M., Day K., Forsyth A., Sallis J.F. (2009). Measuring the Built Environment for Physical Activity: State of the Science. Am. J. Prev. Med..

[12] Tribby C.P., Miller H.J., Brown B.B., Werner C.M., Smith K.R. (2016). Analyzing walking route choice through built environments using random forests and discrete choice techniques. Environ. Plan. B Plan. Des..

[13] Bruce A.F., Theeke L., Mallow J. (2017). A state of the science on influential factors related to sun protective behaviors to prevent skin cancer in adults. Int. J. Nurs. Sci..

[14] Heckman C.J., Manne S.L., Kloss J.D., Bass S.B., Collins B., Lessin S.R. (2011). Beliefs and intentions for skin protection and UV exposure in young adults. Am. J. Health Behav..

[15] Kasparian N.A., McLoone J.K., Meiser B. (2009). Skin cancer-related prevention and screening behaviors: A review of the literature. J. Behav. Med..

[16] (2012). Centers for Disease Control and Prevention (CDC) Sunburn and sun protective behaviors among adults aged 18–29 years--United States, 2000–2010. MMWR Morb. Mortal. Wkly. Rep..

[17] Holman D.M., Berkowitz Z., Guy G.P., Hawkins N.A., Saraiya M., Watson M. (2015). Patterns of sunscreen use on the face and other exposed skin among US adults. J. Am. Acad. Dermatol..

[18] Holman D.M., Berkowitz Z., Guy G.P., Hartman A.M., Perna F.M. (2014). The association between demographic and behavioral characteristics and sunburn among U.S. adults—National Health Interview Survey, 2010. Prev. Med..

[19] National Center for Health Statistics (2016). Survey Description, National Health Interview Survey, 2015.

[20] Paul P., Carlson S.A., Carroll D.D., Berrigan D., Fulton J.E. (2015). Walking for Transportation and Leisure among U.S. Adults—National Health Interview Survey 2010. J. Phys. Act. Health.

[21] Physical Activity Guidelines Advisory Committee (2008). Physical activity guidelines advisory committee report, 2008. Wash. DC US Dep. Health Hum. Serv..

[22] Lawler S., Sugiyama T., Owen N. (2007). Sun exposure concern, sun protection behaviors and physical activity among Australian adults. Cancer Causes Control.

[23] Hamant E.S., Adams B.B. (2005). Sunscreen use among collegiate athletes. J. Am. Acad. Dermatol..

[24] O’Riordan D.L., Steffen A.D., Lunde K.B., Gies P. (2008). A Day at the Beach While on Tropical Vacation: Sun Protection Practices in a High-Risk Setting for UV Radiation Exposure. Arch. Dermatol..

[25] Walkosz B.J., Scott M.D., Buller D.B., Andersen P.A., Beck L., Cutter G.R. (2017). Prevalence of Sun Protection at Outdoor Recreation and Leisure Venues at Resorts in North America. Am. J. Health Educ..

[26] Moehrle M. (2008). Outdoor sports and skin cancer. Clin. Dermatol..

[27] National Institute for Occupational Safety and Health (2018). NIOSH Fast Facts: Protecting Yourself from Sun Exposure.

[28] Linos E., Keiser E., Fu T., Colditz G., Chen S., Tang J.Y. (2011). Hat, shade, long sleeves, or sunscreen? Rethinking US sun protection messages based on their relative effectiveness. Cancer Causes Control.

[29] Lucas R.M., Neale R.E., Madronich S., McKenzie R.L. (2018). Are current guidelines for sun protection optimal for health? Exploring the evidence. Photochem. Photobiol. Sci..

[30] Saraiya M., Glanz K., Briss P.A., Nichols P., White C., Das D., Smith S.J., Tannor B., Hutchinson A.B., Wilson K.M. (2004). Interventions to prevent skin cancer by reducing exposure to ultraviolet radiation: A systematic review. Am. J. Prev. Med..

[31] Autier P., Boniol M., Doré J.-F. (2007). Sunscreen use and increased duration of intentional sun exposure: Still a burning issue. Int. J. Cancer.

[32] Diffey B. (2009). Sunscreens: Expectation and realization. Photodermatol. Photoimmunol. Photomed..

[33] Watts C.G., Drummond M., Goumas C., Schmid H., Armstrong B.K., Aitken J.F., Jenkins M.A., Giles G.G., Hopper J.L., Mann G.J. (2018). Sunscreen Use and Melanoma Risk Among Young Australian Adults. JAMA Dermatol..

[34] Køster B., Thorgaard C., Philip A., Clemmensen I.H. (2010). Prevalence of sunburn and sun-related behaviour in the Danish population: A cross-sectional study. Scand. J. Public Health.

[35] Bennett G.G., Wolin K.Y., Puleo E.M., Mâsse L.C., Atienza A.A. (2009). Awareness of National Physical Activity Recommendations for Health Promotion among US Adults. Med. Sci. Sports Exerc..

[36] Glanz K., Silverio R., Farmer A. (1996). Diary reveals sun protective practices. Skin Cancer Found. J..

[37] Glanz K., Lew R.A., Song V., Cook V.A. (1999). Factors Associated with Skin Cancer Prevention Practices in a Multiethnic Population. Health Educ. Behav..

[38] Manne S.L., Coups E.J., Jacobsen P.B., Ming M., Heckman C.J., Lessin S. (2011). Sun protection and sunbathing practices among at-risk family members of patients with melanoma. BMC Public Health.

[39] Day A.K., Wilson C.J., Hutchinson A.D., Roberts R.M. (2014). The role of skin cancer knowledge in sun-related behaviours: A systematic review. J. Health Psychol..

[40] Morris K.L., Perna F.M. (2018). Decision Tree Model vs Traditional Measures to Identify Patterns of Sun-Protective Behaviors and Sun Sensitivity Associated with Sunburn. JAMA Dermatol..

[41] Bauer J., Büttner P., Wiecker T.S., Luther H., Garbe C. (2005). Interventional study in 1232 young German children to prevent the development of melanocytic nevi failed to change sun exposure and sun protective behavior. Int. J. Cancer.

[42] Jackson K.M., Aiken L.S. (2006). Evaluation of a multicomponent appearance-based sun-protective intervention for young women: Uncovering the mechanisms of program efficacy. Health Psychol..

[43] Sandhu P.K., Elder R., Patel M., Saraiya M., Holman D.M., Perna F., Smith R.A., Buller D., Sinclair C., Reeder A. (2016). Community-wide Interventions to Prevent Skin Cancer: Two Community Guide Systematic Reviews. Am. J. Prev. Med..

[44] Rodrigues A., Sniehotta F.F., Araujo-Soares V. (2013). Are Interventions to Promote Sun-Protective Behaviors in Recreational and Tourist Settings Effective? A Systematic Review with Meta-analysis and Moderator Analysis. Ann. Behav. Med..

[45] Parisi A.V., Turnbull D.J. (2014). Shade Provision for UV Minimization: A Review. Photochem. Photobiol..

[46] Kapelos G.T., Patterson M.R.S. (2014). Health, Planning, Design, And Shade: A Critical Review. J. Archit. Plan. Res..

[47] Culos-Reed S.N., Stephenson L., Doyle-Baker P.K., Dickinson J.A. (2008). Mall Walking as a Physical Activity Option: Results of a Pilot Project. Can. J. Aging Rev. Can. Vieil..

[48] Prince S.A., Adamo K.B., Hamel M.E., Hardt J., Gorber S.C., Tremblay M. (2008). A comparison of direct versus self-report measures for assessing physical activity in adults: A systematic review. Int. J. Behav. Nutr. Phys. Act..

[49] Glanz K., McCarty F., Nehl E.J., O’Riordan D.L., Gies P., Bundy L., Locke A.E., Hall D.M. (2009). Validity of Self-Reported Sunscreen Use by Parents, Children, and Lifeguards. Am. J. Prev. Med..

[50] Dobbinson S.J., Jamsen K., Dixon H.G., Spittal M.J., Lagerlund M., Lipscomb J.E., Herd N.L., Wakefield M.A., Hill D.J. (2014). Assessing population-wide behaviour change: Concordance of 10-year trends in self-reported and observed sun protection. Int. J. Public Health.

[51] O’Riordan D.L., Nehl E., Gies P., Bundy L., Burgess K., Davis E., Glanz K. (2009). Validity of covering-up sun-protection habits: Association of observations and self-report. J. Am. Acad. Dermatol..

[52] Schisterman E.F., Cole S.R., Platt R.W. (2009). Overadjustment Bias and Unnecessary Adjustment in Epidemiologic Studies. Epidemiol. Camb. Mass.

