# Supplementary material for: Walking and Sun Protective Behaviors: Cross-Sectional Associations of Beneficial Health Factors
Source: Int J Environ Res Public Health. 2019 Jul 3;16(13):2361. doi: 10.3390/ijerph16132361 (PMC6651436; doi:10.3390/ijerph16132361)
Supplement: Supplementary file 1 [file ijerph-16-02361-s001.pdf]

Electronic Supplementary Material

Supplement Table 1. Weighted unadjusted and adjusted proportion of US adults and sun-sensitive individuals[a] aged ≥ 18 years who reported regular sunscreen use[b] by exclusive walking category[c] and covariates, National Health Interview Survey, 2015

	Overall, % sunscreen use (95 % CI)					Sun-sensitive, % sunscreen use (95 % CI)		
	Unadjusted	Adjusted, without physical activity level	*P*	Adjusted	*P*	Unadjusted	Adjusted	*P*
Gender			<0.0001		<0.0001			<0.0001
Women	42.5 (41.2-43.8)	41.0 (39.7-42.3)		41.2 (39.9-42.6)		54.1 (52.5-55.8)	52.5 (50.7-54.2)	
Men	22.7 (21.7-23.7)	24.2 (23.1-25.2)		24.0 (23.0-25.1)		32.2 (30.8-33.7)	34.1 (32.7-35.6)	
Walking			<0.0001[d]		<0.0001[e]			<0.0001[f]
No reported walking	23.8 (22.6-25.1)	28.3 (27.0-29.7)		29.6 (28.3-31.0)		34.3 (32.4-36.3)	40.1 (38.1-42.2)	
Transportation walking only	29.5 (27.1-31.8)	31.3 (29.1-33.7)		31.8 (29.5-34.1)		31.0 (37.6-44.4)	43.6 (40.4-46.8)	
Leisure walking only	39.2 (37.7-40.7)	34.8 (33.5-36.1)		34.0 (32.7-35.4)		49.4 (47.5-51.3)	45.3 (43.5-47.1)	
Both transportation and leisure walking	39.5 (37.7-41.3)	36.9 (35.3-38.5)		35.7 (34.2-37.3)		50.8 (48.5-53.2)	47.1 (45.0-49.2)	
Needs walking assistance			<0.0001		<0.0001			<0.0001
Yes	17.5 (15.3-19.8)	23.3 (20.7-26.1)		24.7 (22.1-27.6)		26.2 (22.3-30.0)	33.2 (29.0-37.8)	
No	33.6 (32.7-34.5)	33.2 (32.4-34.1)		33.2 (32.3-34.0)		44.8 (43.6-46.0)	44.4 (43.2-45.6)	
Age group			<0.0001		<0.0001			<0.0001
18-24	25.3 (22.5-28.2)	30.8 (28.0-33.7)		30.4 (27.7-33.3)		34.9 (30.5-39.2)	41.2 (36.9-45.6)	
25-34	32.5 (30.7-34.2)	32.7 (31.0-34.4)		32.5 (30.8-34.2)		43.0 (40.4-45.6)	43.1 (40.6-45.6)	
35-44	38.1 (36.2-40.1)	37.9 (36.2-39.6)		37.8 (36.1-39.5)		49.7 (47.0-52.4)	49.5 (47.1-52.0)	
45-64	34.7 (33.2-36.2)	33.5 (32.1-34.8)		33.6 (32.3-35.0)		46.0 (44.1-47.9)	44.8 (43.0-46.7)	
≥65	28.7 (27.0-30.4)	27.4 (25.6-29.3)		27.7 (25.9-29.6)		40.1 (37.7-42.5)	38.4 (35.6-41.2)	
Race/Ethnicity			<0.0001		<0.0001			0.0008
White, non-Hispanic	39.4 (38.3-40.4)	36.3 (35.2-37.4)		36.2 (35.1-37.3)		46.5 (45.2-47.8)	44.7 (43.4-46.1)	
Black, non-Hispanic	9.6 (8.4-10.9)	14.4 (12.7-16.3)		14.5 (12.8-16.4)		26.6 (20.8-32.4)	32.6 (27.0-38.6)	
Hispanic	23.2 (21.4-25.0)	29.9 (27.8-32.2)		30.0 (27.9-32.3)		31.6 (28.6-34.5)	41.3 (37.8-44.9)	
Other race	28.4 (25.8-31.0)	29.2 (26.8-31.7)		29.3 (26.9-31.8)		37.3 (32.2-42.4)	40.8 (36.1-45.8)	
Education			<0.0001		<0.0001			<0.0001
Less than high school graduate	14.4 (12.7-16.2)	21.5 (19.3-23.8)		22.1 (19.9-24.5)		23.4 (20.0-26.8)	32.8 (28.6-37.3)	
High school graduate	22.4 (21.0-23.8)	27.0 (25.6-28.5)		27.4 (26.0-28.9)		31.1 (29.0-33.3)	36.7 (34.6-38.9)	
Some college	30.1 (28.3-32.0)	32.0 (30.3-33.7)		32.0 (30.3-33.8)		39.6 (36.9-42.3)	42.0 (39.6-44.5)	
College graduate	44.1 (42.8-45.4)	38.0 (36.8-39.3)		37.6 (36.3-38.9)		54.4 (52.5-56.1)	49.1 (47.4-50.8)	
Marital status			<0.0001		<0.0001			0.005
Married or living with	36.5 (35.3-37.6)	34.0 (32.9-35.1)		34.0 (32.9-35.1)		46.8 (45.3-48.3)	45.1 (43.6-46.6)	

partner								
Divorced or separated	30.3 (28.5-32.2)	32.2 (30.6-34.0)		32.3 (30.7-34.1)		43.2 (40.5-46.0)	44.1 (41.6-46.7)	
Widowed	26.4 (23.9-29.0)	29.2 (26.5-32.0)		29.4 (26.6-32.2)		38.0 (34.0-42.0)	39.9 (35.7-44.4)	
Never married	24.9 (23.2-26.6)	29.9 (28.3-31.6)		29.8 (28.2-31.5)		36.0 (33.4-38.6)	40.6 (38.1-43.0)	
Foreign born status			0.14		0.22			0.03
United States born	34.3 (33.4-35.2)	33.1 (32.2-34.0)		33.0 (32.1-34.0)		45.1 (43.8-46.4)	44.5 (43.2-45.7)	
<10 y in the United States	24.9 (21.0-28.7)	30.4 (26.8-34.2)		30.9 (27.3-34.7)		31.0 (24.4-37.7)	38.7 (32.5-45.3)	
≥10 y in the United States	25.6 (23.8-27.5)	31.2 (29.0-33.4)		31.3 (29.2-33.5)		35.5 (32.1-38.9)	39.4 (35.7-43.3)	
US Census region			0.0012		0.003			0.24
Northeast	36.1 (33.8-38.4)	34.4 (32.3-36.5)		34.5 (32.4-36.6)		48.0 (45.0-51.1)	45.9 (43.3-48.5)	
Midwest	30.3 (28.8-31.8)	30.2 (28.9-31.6)		30.4 (29.0-31.8)		40.9 (38.8-43.1)	42.5 (40.5-44.6)	
South	30.5 (29.1-31.9)	32.9 (31.6-34.2)		32.9 (31.6-34.2)		43.2 (41.2-45.2)	43.8 (41.9-45.6)	
West	35.7 (33.9-37.6)	33.6 (32.0-35.2)		33.4 (31.8-35.0)		44.5 (42.1-46.9)	43.8 (41.6-46.0)	
Sunburn in past 12 months			<0.0001		<0.0001			0.08
Yes	42.1 (40.7-43.5)	35.9 (34.7-37.2)		35.7 (34.5-37.0)		46.3 (44.7-48.0)	44.8 (43.2-46.3)	
No	27.4 (26.4-28.3)	30.7 (29.6-31.7)		30.8 (29.7-31.8)		41.2 (39.6-42.7)	42.9 (41.3-44.5)	
Skin reaction after 2 weeks in sun			<0.0001		<0.0001			<.0001
Very dark	22.8 (20.6-25.0)	28.9 (26.7-31.1)		28.7 (26.5-30.9)		32.3 (28.1-36.6)	38.1 (34.3-42.1)	
Moderate tan	28.8 (27.4-30.2)	30.4 (29.1-31.7)		30.2 (28.9-31.6)		36.5 (34.4-38.7)	38.8 (36.9-40.9)	
Mild tan	31.8 (30.4-33.2)	32.2 (30.9-33.5)		32.2 (31.0-33.5)		41.6 (39.6-43.6)	42.3 (40.4-44.2)	
Burn repeatedly or freckle	54.5 (52.4-56.5)	43.7 (41.9-45.5)		43.7 (41.9-45.6)		58.6 (56.4-60.8)	53.4 (51.3-55.5)	
Do not go out in the sun	18.4 (16.0-20.8)	22.1 (19.6-24.9)		22.8 (20.2-25.5)		40.6 (33.0-48.2)	40.1 (32.9-47.7)	
Regular sun avoidance[g]			<0.0001		<0.0001			<.0001
Yes	36.8 (35.5-38.0)	37.3 (36.0-38.5)		37.5 (36.3-38.8)		40.0 (38.4-41.5)	48.7 (46.9-50.5)	
No	29.8 (28.8-30.9)	29.7 (28.7-30.8)		29.6 (28.6-30.6)		49.3 (47.5-51.1)	40.5 (39.0-42.0)	
Regular sun protective clothing use[h]			<0.0001		<0.0001			0.15
Yes	34.2 (32.9-35.5)	35.2 (34.0-36.4)		35.2 (34.0-36.4)		43.6 (41.8-45.5)	44.8 (43.1-46.6)	
No	31.8 (30.7-32.9)	31.4 (30.4-32.4)		31.4 (30.4-32.4)		43.9 (42.5-45.4)	43.3 (41.9-44.7)	
Physical activity level[i]			-		<0.0001			<.0001
Inactive	20.9 (19.5-22.2)	-		27.9 (26.4-29.5)		31.9 (29.6-34.2)	39.4 (37.0-41.8)	
Insufficiently active	32.2 (30.4-34.0)	-		30.7 (29.2-32.3)		41.1 (38.8-43.5)	41.0 (38.8-43.2)	
Sufficiently active	39.2 (37.4-41.0)	-		34.6 (33.0-36.3)		50.8 (48.3-53.2)	46.4 (44.2-48.6)	
Highly active	39.3 (38.0-40.7)	-		36.2 (35.0-37.5)		50.0 (48.1-52.0)	47.2 (45.3-49.0)	
Obesity or overweight			0.0009		0.005			0.02
Yes	30.1 (29.1-31.1)	31.7 (30.7-32.8)		31.9 (30.8-32.9)		41.2 (39.7-42.7)	42.8 (41.3-44.3)	
No	37.1 (35.6-38.5)	34.4 (33.1-35.8)		34.2 (32.9-35.5)		48.3 (46.3-50.3)	45.7 (43.8-47.5)	
Smoking status			<0.0001		<0.0001			<0.0001
Never	35.6 (34.5-36.7)	34.7 (33.6-35.7)		34.5 (33.5-35.6)		47.7 (46.2-49.2)	46.1 (44.6-47.5)	

Former	33.2 (31.5-34.9)	32.5 (30.9-34.1)		32.4 (30.8-34.0)		43.3 (40.9-45.7)	43.6 (41.2-46.1)	
Current	19.1 (17.6-20.7)	23.8 (22.0-25.7)		24.3 (22.5-26.2)		27.1 (24.8-29.5)	33.5 (30.8-36.3)	
Binge drinking in past 30 d			0.01		0.04			0.07
Yes	32.9 (30.7-35.0)	35.1 (33.1-37.2)		34.7 (32.7-36.8)		41.8 (39.0-44.7)	46.1 (42.5-48.8)	
No	32.6 (31.7-33.5)	32.3 (31.4-33.2)		32.4 (31.5-33.3)		44.2 (42.9-45.5)	43.4 (42.2-44.7)	
Ever had a skin exam			<0.0001		<0.0001			<0.0001
Yes	41.4 (49.6-53.1)	41.3 (39.7-42.9)		41.0 (39.4-42.6)		59.6 (57.6-61.6)	52.5 (50.5-54.5)	
No	27.0 (26.1-27.9)	29.8 (28.9-30.8)		29.9 (29.0-30.9)		37.3 (35.9-38.7)	40.2 (38.8-41.6)	
Personal history of melanoma			0.06		0.05			0.74
Yes	54.4 (44.9-63.9)	40.8 (32.5-49.8)		41.0 (32.5-50.0)		58.2 (47.6-68.7)	45.6 (35.5-56.2)	
No	32.5 (31.7-33.4)	32.7 (31.8-33.5)		32.7 (31.8-33.5)		43.7 (42.6-44.9)	43.8 (42.7-45.0)	
Family history of melanoma			0.31		0.38			0.75
Yes	48.1 (43.0-53.1)	34.9 (30.7-39.3)		34.6 (30.4-39.0)		50.7 (45.1-56.3)	43.0 (37.9-48.3)	
No	32.3 (31.4-33.1)	32.7 (31.8-33.5)		32.7 (31.8-33.5)		43.6 (42.4-44.8)	43.9 (42.7-45.1)	
Ever had cancer			0.08		0.08			0.10
Yes	40.5 (37.8-43.1)	31.7 (32.3-37.3)		34.8 (32.3-37.3)		50.9 (47.3-54.4)	46.6 (43.1-50.1)	
No	32.0 (31.1-32.8)	32.5 (31.7-33.4)		32.5 (31.7-33.4)		43.1 (41.8-44.3)	43.6 (42.3-44.8)	
Indoor tanning			0.01		0.008			0.04
Yes	35.6 (31.5-39.7)	28.3 (25.1-31.8)		28.2 (25.0-31.7)		41.3 (36.1-46.5)	38.9 (34.0-44.0)	
No	32.6 (31.7-33.4)	32.9 (32.1-33.8)		32.9 (32.1-33.8)		44.0 (42.8-45.1)	44.1 (42.9-45.3)	
Sunless tanning (self-applied)			0.05		0.07			0.20
Yes	55.1 (51.4-58.7)	35.5 (32.6-38.5)		35.3 (32.4-38.3)		59.7 (55.6-63.7)	46.2 (42.4-50.0)	
No	31.4 (30.5-32.2)	32.5 (31.7-33.4)		32.6 (31.7-33.4)		42.5 (41.2-43.7)	43.6 (42.4-44.9)	
Insurance status			0.01		0.02			0.05
Uninsured	20.3 (18.3-22.4)	29.9 (27.6-32.4)		30.0 (27.7-32.5)		28.9 (25.5-32.3)	40.4 (36.7-44.2)	
Insured	34.2 (33.3-35.1)	33.0 (32.1-33.9)		33.0 (32.1-33.8)		45.2 (44.0-46.4)	44.4 (43.2-45.6)	

[a]Sun-sensitivity defined as reporting any skin burn when not protected from the sun for 1 hour.

[b]Regular sunscreen use was defined as always or usually using sunscreen with SPF15+ (sun protection factor).

[c]Reported walking categories for at least 10 minutes in the past 7 days.

[d]Overall significant contrasts, without leisure-time physical activity covariate: no reported walking vs both, p<0.0001; no reported walking vs transportation, p=0.02; no reported walking vs leisure, p<0.0001; transportation vs both, p<0.0001; leisure vs both, p=0.04; transportation vs leisure, p=0.02.

[e]Overall significant contrasts: no reported walking vs both, p<0.0001; no reported walking vs leisure, p<0.0001; transportation vs both, p=0.003.

[g]Sun avoidance was defined as always or usually staying in the shade or responding not going into the sun for any sun protective question.

[h]Sun protective clothing was defined as always or usually wearing at least 1: wide-brimmed hat, long sleeved shirt, or long clothing to the ankles.

[i]Individuals were categorized into 4 activity levels based on the 2008 Physical Activity Guidelines for Americans: highly active (>300 min/week of light or moderate-intensity aerobic activity, 150 minutes of vigorous-intensity aerobic activity, or an equivalent combination per week [i.e., moderate-intensity equivalent activity]), sufficiently active (150–300 min/week of moderate-intensity equivalent activity), insufficiently active (some activity but less than 150 min/week of moderate-intensity equivalent activity), and inactive (no light to moderate or vigorous-intensity aerobic activity for at least 10 minutes).

[f]Sun-sensitive significant contrasts: no reported walking vs both, p<0.0001; no reported walking vs leisure, p=0.0001.

Supplement Table 2. Weighted unadjusted and adjusted proportion of US adults and sun-sensitive individuals[a] aged ≥ 18 years who reported regular sun avoidance[b] by exclusive walking category[c] and covariates, National Health Interview Survey, 2015

	Overall, % sun avoidance (95 % CI)					Sun-sensitive, % sun avoidance (95 % CI)		
	Unadjusted	Adjusted, without physical activity level	*P*	Adjusted	*P*	Unadjusted	Adjusted	*P*
Gender			<0.0001		<0.0001			<0.0001
Women	48.3 (47.2-49.4)	46.9 (45.8-48.0)		46.5 (45.4-47.7)		47.4 (46.0-48.9)	46.2 (44.8-47.8)	
Men	33.4 (32.2-34.7)	34.9 (33.7-36.2)		35.2 (34.0-36.5)		34.5 (32.7-36.3)	35.8 (34.0-37.6)	
Walking			<0.0001[d]		0.03[e]			0.04[f]
No reported walking	45.0 (43.6-46.4)	42.9 (41.5-44.3)		42.0 (40.6-43.5)		45.0 (42.8-47.2)	42.5 (40.3-44.7)	
Transportation walking only	41.9 (39.4-44.4)	42.6 (40.3-45.0)		42.3 (40.0-44.6)		44.0 (40.3-47.6)	44.6 (41.3-47.9)	
Leisure walking only	39.0 (37.6-40.4)	40.1 (38.7-41.5)		40.5 (39.2-41.9)		39.6 (37.7-41.5)	40.3 (38.5-42.1)	
Both transportation and leisure walking	36.2 (34.4-38.0)	37.9 (36.2-39.6)		38.9 (37.2-40.6)		37.4 (34.9-39.9)	39.8 (37.4-42.3)	
Needs walking assistance			<0.0001		<0.0001			<0.0001
Yes	65.1 (62.2-67.9)	53.4 (50.3-56.5)		52.3 (49.1-55.4)		66.7 (62.6-70.8)	54.8 (50.2-59.3)	
No	39.4 (38.5-40.3)	40.2 (39.3-41.1)		40.2 (39.3-41.1)		40.0 (38.7-41.3)	40.6 (39.3-41.9)	
Age group			0.02		0.06			0.13
18-24	33.6 (30.9-36.2)	37.1 (34.3-39.9)		37.6 (34.8-40.4)		31.9 (28.1-35.7)	36.5 (32.5-40.8)	
25-34	36.2 (34.4-37.9)	40.0 (38.1-41.8)		40.2 (38.4-42.0)		36.6 (34.3-38.8)	41.2 (38.7-43.7)	
35-44	38.6 (36.6-40.5)	40.3 (38.4-42.2)		40.3 (38.5-42.2)		40.0 (37.1-42.8)	42.1 (39.4-44.9)	
45-64	42.1 (40.5-43.6)	42.0 (40.5-43.5)		41.7 (40.3-43.2)		42.9 (40.8-44.9)	41.9 (40.0-43.9)	
≥65	52.2 (50.4-54.0)	43.4 (41.3-45.4)		43.1 (41.1-45.2)		52.3 (49.7-54.8)	43.0 (40.2-45.8)	
Race/Ethnicity			<0.0001		<0.0001			<0.0001
White, non-Hispanic	36.3 (35.2-37.3)	37.3 (36.2-38.4)		37.3 (36.2-38.4)		38.7 (37.3-40.1)	39.8 (38.4-41.3)	
Black, non-Hispanic	53.3 (50.8-55.7)	51.1 (48.5-53.7)		51.0 (48.4-53.6)		56.5 (50.4-62.6)	53.3 (47.5-59.1)	
Hispanic	49.3 (47.3-51.4)	47.0 (44.8-49.3)		47.0 (44.7-49.2)		55.3 (51.7-58.9)	48.1 (44.3-51.9)	
Other race	45.0 (42.4-47.7)	43.8 (41.0-46.7)		43.6 (40.8-46.5)		46.4 (41.4-51.5)	43.9 (39.1-48.7)	
Education			0.01		0.03			0.11
Less than high school graduate	48.0 (45.6-50.4)	41.0 (38.8-43.3)		40.6 (38.4-42.8)		49.9 (45.7-54.0)	41.4 (37.6-45.3)	
High school graduate	41.8 (40.1-43.6)	41.4 (39.7-43.1)		41.1 (39.4-42.8)		44.2 (41.7-46.7)	43.4 (41.1-45.8)	
Some college	41.5 (39.6-43.4)	42.9 (41.2-47.7)		42.9 (41.1-44.6)		41.3 (38.9-43.7)	42.2 (40.0-44.5)	
College graduate	38.3 (37.1-39.5)	38.7 (38.5-41.0)		40.0 (38.8-41.3)		38.8 (37.1-40.4)	40.2 (38.4-41.9)	
Marital status			0.0001		0.0001			0.003

Married or living with partner	40.0 (38.8-41.2)	40.4 (39.2-41.6)		40.3 (39.2-41.5)		41.1 (39.5-42.7)	40.8 (39.2-42.4)	
Divorced or separated	42.6 (40.5-44.7)	38.4 (36.5-40.3)		38.4 (36.5-40.4)		42.2 (39.3-45.2)	38.3 (35.6-41.1)	
Widowed	60.3 (57.5-63.1)	44.5 (41.4-47.7)		44.5 (41.4-47.7)		59.2 (54.8-63.7)	45.1 (40.3-50.0)	
Never married	38.2 (36.4-40.0)	42.8 (41.0-44.6)		42.9 (41.1-44.8)		37.9 (35.3-40.4)	44.2 (41.8-46.7)	
Foreign born status			0.003		0.004			0.0001
United States born	38.7 (37.7-39.7)	40.1 (39.1-41.1)		40.1 (39.1-41.1)		39.4 (38.0-40.7)	40.4 (39.0-41.7)	
<10 y in the United States	46.8 (42.8-51.1)	43.8 (39.7-48.0)		43.2 (39.1-47.5)		50.0 (42.7-57.3)	46.4 (39.6-53.4)	
≥10 y in the United States	51.6 (49.4-53.7)	44.4 (42.1-46.7)		44.3 (42.0-46.7)		57.5 (53.9-61.0)	48.9 (45.1-52.7)	
US Census region			<0.0001		<0.0001			<0.0001
Northeast	39.0 (36.9-41.2)	40.0 (38.0-42.1)		39.9 (37.9-42.0)		40.5 (37.4-43.7)	41.3 (38.4-44.2)	
Midwest	30.2 (28.6-31.7)	34.1 (32.5-35.6)		33.9 (32.4-35.5)		31.2 (28.8-33.7)	34.3 (32.0-36.7)	
South	46.2 (44.6-47.8)	43.8 (42.3-45.3)		43.9 (42.4-45.3)		45.0 (42.6-47.4)	44.0 (41.9-46.2)	
West	44.2 (42.4-46.0)	43.1 (41.3-44.9)		43.2 (41.5-45.1)		46.4 (44.1-48.7)	44.1 (41.9-46.3)	
Sunburn in past 12 months			<0.0001		<0.0001			<0.0001
Yes	32.7 (31.3-34.0)	38.1 (36.7-39.6)		38.2 (36.8-39.6)		34.4 (32.8-36.0)	37.7 (36.1-39.3)	
No	45.5 (44.5-46.6)	42.4 (41.3-43.5)		42.4 (41.2-43.5)		48.8 (47.1-50.6)	45.1 (43.2-46.9)	
Skin reaction after 2 weeks in sun			<0.0001		<0.0001			<0.0001
Very dark	35.8 (33.3-38.3)	35.1 (32.7-37.5)		35.3 (32.9-37.7)		40.2 (36.0-44.4)	38.7 (34.6-42.9)	
Moderate tan	32.4 (30.9-33.8)	34.3 (32.9-35.7)		34.5 (33.1-35.9)		34.1 (31.9-36.3)	36.2 (34.2-38.3)	
Mild tan	35.3 (33.8-36.7)	35.6 (34.3-37.0)		35.5 (34.2-36.9)		36.2 (34.2-38.2)	36.8 (34.9-38.6)	
Burn repeatedly or freckle	51.8 (49.7-53.9)	50.7 (48.6-52.8)		50.5 (48.5-52.6)		54.0 (51.7-56.2)	51.5 (49.3-53.7)	
Do not go out in the sun	85.9 (83.8-88.0)	81.2 (78.4-83.8)		80.9 (78.0-83.5)		75.0 (69.8-81.3)	67.3 (60.6-73.4)	
Regular sunscreen use[g]			<0.0001		<0.0001			<0.0001
Yes	46.0 (44.5-47.5)	46.6 (45.1-48.2)		46.9 (45.4-48.5)		46.6 (44.7-48.4)	46.0 (44.1-47.8)	
No	38.4 (37.4-39.5)	38.0 (37.0-39.1)		37.9 (36.8-38.9)		37.3 (35.7-39.0)	37.7 (36.1-39.4)	
Regular sun protective clothing use[h]			<0.0001		<0.0001			<0.0001
Yes	51.6 (50.1-53.0)	50.3 (48.9-51.7)		50.2 (48.8-51.6)		55.4 (53.3-57.4)	52.2 (50.2-54.1)	
No	34.8 (33.9-35.9)	35.4 (34.4-36.4)		37.9 (36.8-38.9)		33.2 (31.7-34.6)	34.9 (33.5-36.4)	
Physical activity level[i]			-		<0.0001			<0.0001
Inactive	49.2 (47.7-50.8)	-		43.4 (41.9-44.9)		49.5 (47.1-51.8)	44.6 (42.3-47.0)	
Insufficiently active	44.7 (42.8-46.6)	-		44.4 (42.7-46.2)		47.0 (44.6-49.4)	45.7 (43.5-48.0)	
Sufficiently active	39.1 (37.1-41.0)	-		40.9 (39.1-42.7)		39.9 (37.1-42.6)	41.0 (38.3-43.7)	
Highly active	32.8 (31.3-34.3)	-		36.7 (35.3-38.2)		33.3 (31.5-35.1)	36.6 (34.7-38.5)	
Obesity or overweight			<0.0001		<0.0001			<0.0001
Yes	42.5 (41.4-43.6)	41.3 (40.4-42.3)		42.7 (41.7-43.7)		43.9 (42.3-45.5)	44.0 (42.5-45.6)	
No	38.1 (36.7-39.5)	38.2 (36.1-40.3)		37.8 (36.4-39.2)		37.0 (35.2-38.8)	36.9 (35.1-38.7)	
Smoking status			0.91		0.83			0.59

Never	42.0 (40.9-43.2)	41.0 (39.9-42.1)		41.1 (40.0-42.2)		42.0 (40.4-43.7)	41.8 (40.2-43.4)	
Former	40.5 (38.9-42.2)	40.6 (39.0-42.3)		40.7 (39.1-42.4)		41.7 (39.5-43.9)	40.5 (38.4-42.6)	
Current	36.5 (34.4-38.6)	40.7 (38.8-42.7)		40.4 (38.4-42.4)		37.9 (34.8-41.0)	40.9 (38.0-43.9)	
Binge drinking in past 30 d			0.006		0.01			0.0009
Yes	28.8 (26.8-30.8)	38.2 (36.1-40.3)		38.5 (36.4-40.6)		28.5 (25.9-31.1)	36.8 (33.9-39.7)	
No	43.0 (42.0-43.9)	41.3 (40.4-42.3)		41.3 (40.3-42.2)		43.9 (42.5-45.3)	42.2 (40.8-43.6)	
Ever had a skin exam			0.01		0.005			0.03
Yes	43.7 (42.0-45.4)	42.7 (41.0-44.4)		42.9 (41.2-44.6)		44.8 (42.6-47.0)	43.2 (41.1-45.2)	
No	40.1 (39.1-41.1)	40.4 (39.4-41.4)		40.3 (39.3-41.3)		40.0 (38.5-41.4)	40.6 (39.1-42.1)	
Personal history of melanoma			0.11		0.11			0.08
Yes	58.1 (47.9-68.4)	49.0 (39.1-58.9)		48.4 (39.1-58.7)		59.7 (48.3-71.1)	51.3 (40.3-62.3)	
No	40.8 (39.9-41.7)	40.9 (39.9-41.8)		40.9 (39.9-41.8)		41.2 (39.9-42.5)	41.3 (40.0-42.6)	
Family history of melanoma			0.79		0.68			0.91
Yes	41.6 (36.9-46.4)	41.5 (37.2-45.9)		41.8 (37.5-46.3)		42.8 (37.4-48.2)	41.7 (36.7-46.8)	
No	40.9 (40.0-41.8)	40.9 (40.0-41.8)		40.9 (40.0-41.8)		41.3 (40.0-42.6)	41.4 (40.1-42.7)	
Ever had cancer			0.80		0.84			0.97
Yes	50.2 (47.3-53.1)	41.2 (38.4-44.1)		41.2 (38.3-44.0)		50.4 (46.6-54.2)	41.4 (37.8-45.2)	
No	40.1 (39.2-41.0)	40.9 (40.0-41.8)		40.9 (40.0-41.8)		40.4 (39.1-41.8)	41.4 (40.1-42.7)	
Indoor tanning			<0.0001		<0.0001			<0.0001
Yes	18.7 (15.4-22.0)	29.2 (25.4-33.3)		29.5 (25.7-33.6)		20.4 (16.2-24.6)	29.7 (25.0-34.9)	
No	41.8 (40.9-42.7)	41.3 (40.4-42.2)		41.3 (40.4-42.2)		42.4 (41.1-43.7)	41.9 (40.5-43.2)	
Sunless tanning (self-applied)			0.41		0.45			0.85
Yes	36.7 (33.4-40.1)	39.6 (36.3-43.0)		39.7 (35.4-43.1)		39.7 (35.6-43.9)	41.7 (37.8-45.7)	
No	41.2 (40.2-42.1)	41.0 (40.1-41.9)		41.0 (40.1-41.9)		41.5 (40.2-42.9)	41.3 (40.0-42.7)	
Insurance status			0.86		0.85			0.18
Uninsured	41.7 (39.2-44.3)	41.1 (38.7-43.6)		41.1 (38.7-43.6)		45.4 (41.6-49.2)	43.9 (40.2-47.6)	
Insured	40.8 (39.8-41.8)	40.9 (40.0-41.9)		40.9 (40.0-41.8)		41.0 (39.6-42.4)	41.1 (39.8-42.5)	

[a]Sun-sensitivity defined as reporting any skin burn when not protected from the sun for 1 hour.

[b]Sun avoidance was defined as always or usually staying in the shade or responding not going into the sun for any sun protective question.

[c]Reported walking categories for at least 10 minutes in the past 7 days.

[d]Overall significant contrasts, without leisure-time physical activity covariate: no reported walking vs both, p<0.0001; no reported walking vs leisure, p=0.003; transportation vs both, p=0.001; leisure vs both, p=0.04.

[e]Overall significant contrasts: no reported walking vs both, p=0.006; transportation vs both, p=0.02.

[f]Sun-sensitive significant contrasts: transportation vs both, p=0.02.

[g]Regular sunscreen use was defined as always or usually using sunscreen with SPF15+ (sun protection factor).

[h]Sun protective clothing was defined as always or usually wearing at least 1: wide-brimmed hat, long sleeved shirt, or long clothing to the ankles.

[i]Individuals were categorized into 4 activity levels based on the 2008 Physical Activity Guidelines for Americans: highly active (>300 min/week of light or moderate-intensity aerobic activity, 150 minutes of vigorous-intensity aerobic activity, or an equivalent combination per week [i.e., moderate-intensity equivalent activity]), sufficiently active (150–300 min/week of moderate-intensity equivalent activity), insufficiently active (some activity but less than 150 min/week of

moderate-intensity equivalent activity), and inactive (no light to moderate or vigorous-intensity aerobic activity for at least 10 minutes).

Supplement Table 3. Weighted and adjusted proportion of US adults and sun-sensitive individuals[a] aged ≥ 18 years who reported regular sun protective clothing use[b] by exclusive walking category[c] and covariates, National Health Interview Survey, 2015

	Overall, % sun protective clothing (95 % CI)					Sun-sensitive, % sun protective clothing (95 % CI)		
	Unadjusted	Adjusted, without physical activity level	*P*	Adjusted	*P*	Unadjusted	Adjusted	*P*
Gender			<0.0001		<0.0001			<0.0001
Women	30.9 (29.9-32.0)	29.8 (28.7-30.8)		29.7 (28.7-30.8)		32.5 (31.0-34.0)	31.4 (29.9-32.9)	
Men	41.7 (40.4-43.0)	43.0 (41.8-44.3)		43.1 (41.8-44.3)		42.1 (40.4-43.8)	43.5 (41.8-45.2)	
Walking			<0.0001[d]		<0.0001[e]			0.04[f]
No reported walking	34.7 (33.4-36.1)	34.3 (33.0-35.6)		34.4 (33.0-35.8)		37.1 (35.2-39.1)	35.6 (33.6-37.7)	
Transportation walking only	37.1 (34.7-39.6)	37.1 (34.8-39.4)		37.1 (34.8-39.4)		38.1 (34.7-41.5)	37.2 (34.0-40.4)	
Leisure walking only	35.7 (34.3-37.0)	35.8 (34.5-37.2)		35.7 (34.4-37.1)		35.3 (33.4-37.2)	36.3 (34.4-38.2)	
Both transportation and leisure walking	39.3 (37.4-41.2)	40.0 (38.2-41.9)		40.0 (38.1-41.9)		39.2 (36.6-41.8)	40.3 (37.8-43.0)	
Needs walking assistance			0.63		0.57			0.74
Yes	42.9 (40.0-45.8)	36.9 (37.1-42.4)		37.0 (34.3-39.9)		48.5 (44.0-53.0)	37.7 (33.6-41.9)	
No	35.8 (34.9-36.7)	36.2 (35.3-37.1)		36.2 (35.3-37.1)		36.4 (35.2-37.5)	37.0 (35.8-38.2)	
Age group			<0.0001		<0.0001			<0.0001
18-24	25.9 (23.4-28.4)	26.8 (24.2-29.7)		26.9 (24.3-29.7)		26.7 (23.0-30.3)	27.2 (23.6-31.2)	
25-34	30.5 (28.6-32.3)	30.5 (28.6-32.4)		30.5 (28.7-32.4)		30.5 (27.9-33.0)	31.3 (28.8-33.9)	
35-44	32.8 (31.1-34.5)	32.4 (30.7-34.1)		32.3 (30.7-34.0)		32.7 (30.3-35.0)	33.0 (30.7-35.4)	
45-64	38.8 (37.4-40.3)	39.0 (37.6-40.4)		39.0 (37.5-40.4)		38.6 (36.8-40.4)	39.3 (37.4-41.2)	
≥65	49.2 (47.4-51.0)	48.4 (46.2-50.6)		48.3 (46.2-50.5)		53.6 (51.0-56.2)	49.7 (46.7-52.7)	
Race/Ethnicity			0.0001		0.0001			0.001
White, non-Hispanic	34.1 (33.0-35.2)	34.6 (33.5-35.8)		34.6 (33.5-35.8)		35.0 (33.7-36.3)	35.8 (34.4-37.1)	
Black, non-Hispanic	35.4 (22.0-37.8)	38.9 (35.4-41.4)		38.9 (36.4-41.4)		40.7 (33.9-47.4)	42.7 (36.8-48.8)	
Hispanic	43.8 (41.8-45.7)	40.0 (37.8-42.2)		40.0 (37.9-42.2)		48.0 (44.8-51.1)	43.3 (39.5-47.1)	
Other race	40.2 (37.6-42.9)	38.4 (35.7-41.2)		38.3 (35.6-41.1)		42.5 (37.8-47.2)	39.2 (34.5-44.1)	
Education			<0.0001		<0.0001			0.02
Less than high school graduate	45.2 (42.7-47.7)	41.2 (38.8-43.6)		41.2 (38.9-43.7)		47.8 (43.8-51.9)	41.6 (37.7-45.6)	
High school graduate	37.6 (36.0-39.3)	38.2 (36.7-39.9)		38.3 (36.7-39.9)		39.4 (37.0-41.9)	38.8 (36.4-41.4)	
Some college	34.2 (32.4-36.1)	36.0 (34.3-37.9)		36.0 (34.3-37.8)		35.1 (32.7-37.6)	36.3 (34.1-38.5)	
College graduate	34.0 (32.9-35.2)	34.0 (32.7-35.2)		34.0 (32.7-35.2)		34.9 (33.4-36.3)	35.7 (34.2-37.3)	
Marital status			0.31		0.31			0.02
Married or living with	37.4 (36.2-38.6)	36.3 (35.1-37.6)		36.3 (35.1-37.5)		37.1 (35.7-38.5)	36.4 (34.9-37.8)	

partner								
Divorced or separated	36.0 (33.9-38.1)	34.8 (32.9-36.8)		34.8 (32.9-36.8)		36.3 (33.5-39.1)	34.7 (32.0-37.5)	
Widowed	45.8 (43.0-48.6)	38.0 (35.1-41.1)		38.1 (35.1-41.1)		51.8 (47.6-56.1)	39.3 (35.1-43.8)	
Never married	31.1 (29.4-32.8)	36.4 (34.6-38.3)		36.4 (34.6-38.3)		33.9 (31.3-36.5)	40.0 (37.3-42.8)	
Foreign born status			<0.0001		<0.0001			0.004
United States born	34.4 (33.4-35.4)	35.3 (34.3-36.3)		35.3 (34.3-36.3)		35.3 (34.1-36.6)	36.2 (35.0-37.4)	
<10 y in the United States	42.6 (38.6-46.5)	44.3 (40.1-48.6)		44.3 (40.1-48.6)		46.5 (39.3-53.6)	45.4 (37.3-53.9)	
≥10 y in the United States	44.8 (42.8-46.9)	39.3 (37.2-41.5)		39.4 (37.2-41.5)		49.9 (46.5-53.2)	42.1 (38.4-45.9)	
US Census region			<0.0001		<0.0001			<0.0001
Northeast	28.7 (26.8-30.6)	28.4 (26.7-30.2)		28.5 (26.8-30.3)		29.1 (25.5-31.8)	28.8 (26.4-31.3)	
Midwest	31.8 (29.9-33.7)	34.8 (32.9-36.8)		34.8 (32.8-36.7)		32.6 (30.2-34.9)	35.9 (33.6-38.4)	
South	36.2 (34.8-37.6)	38.0 (35.1-41.1)		35.9 (34.5-37.4)		37.4 (35.4-39.3)	36.9 (35.1-38.7)	
West	45.6 (43.8-47.4)	43.5 (41.7-45.3)		43.5 (41.6-45.3)		45.9 (43.6-48.2)	43.6 (41.3-45.9)	
Sunburn in past 12 months			0.40		0.38			0.83
Yes	32.0 (30.6-33.4)	35.8 (34.3-37.2)		35.7 (34.3-37.2)		32.5 (31.0-34.0)	36.9 (35.3-38.5)	
No	38.6 (37.5-39.7)	36.5 (35.4-37.7)		36.6 (35.4-37.7)		41.8 (40.1-43.6)	37.1 (35.4-38.9)	
Skin reaction after 2 weeks in sun			<0.0001		<0.0001			0.0003
Very dark	36.9 (34.6-39.3)	37.0 (34.9-39.2)		40.0 (34.8-39.1)		37.5 (33.6-41.4)	35.3 (31.7-39.0)	
Moderate tan	34.7 (33.3-36.1)	35.6 (34.3-37.1)		35.6 (34.3-37.1)		34.7 (32.7-36.7)	35.3 (33.3-37.3)	
Mild tan	36.2 (34.7-37.6)	37.5 (36.1-38.8)		37.4 (36.1-38.8)		34.5 (32.8-36.3)	35.6 (34.0-37.3)	
Burn repeatedly or freckle	41.8 (39.5-44.1)	42.0 (39.8-44.2)		42.0 (39.8-44.2)		42.0 (39.6-44.4)	41.3 (39.1-43.7)	
Do not go out in the sun	28.8 (26.1-31.6)	21.3 (19.0-23.7)		21.4 (19.2-23.9)		44.1 (36.8-41.4)	35.1 (28.9-41.9)	
Regular sunscreen use[g]			<0.0001		<0.0001			0.13
Yes	37.9 (36.5-39.4)	39.4 (38.0-40.8)		39.4 (38.0-40.8)		36.8 (35.1-38.6)	37.9 (36.3-39.6)	
No	35.4 (34.4-36.4)	34.8 (33.8-35.8)		34.8 (33.8-35.8)		37.1 (35.7-38.6)	36.3 (34.9-37.7)	
Regular sun avoidance[h]			<0.0001		<0.0001			<0.0001
Yes	45.7 (44.3-47.0)	45.5 (44.1-46.9)		45.5 (44.0-46.9)		49.5 (47.7-51.3)	47.2 (45.4-49.0)	
No	29.7 (28.7-30.7)	29.9 (28.9-30.9)		29.9 (28.9-30.9)		28.2 (26.8-29.6)	29.6 (28.2-31.0)	
Physical activity level[i]			-		0.12			0.66
Inactive	36.5 (35.0-38.0)	-		35.4 (33.8-37.1)		40.0 (37.8-42.2)	36.9 (34.5-39.4)	
Insufficiently active	39.0 (37.2-40.8)	-		38.2 (36.4-40.0)		39.4 (37.0-41.7)	38.1 (36.0-40.4)	
Sufficiently active	35.2 (33.2-37.3)	-		35.8 (33.9-37.8)		34.9 (32.2-37.5)	36.2 (33.6-38.8)	
Highly active	34.9 (33.5-36.3)	-		36.0 (34.6-37.5)		34.6 (32.8-36.4)	36.8 (34.9-38.6)	
Obesity or overweight			0.26		0.25			0.30
Yes	34.4 (33.0-35.7)	35.9 (34.9-37.0)		35.9 (34.8-37.0)		38.2 (36.8-39.6)	37.8 (35.9-39.7)	
No	37.3 (36.2-38.4)	36.9 (35.5-39.3)		36.9 (35.6-38.3)		35.0 (33.1-36.9)	36.6 (35.2-38.0)	
Smoking status			0.04		0.04			0.009
Never	34.6 (33.7-35.7)	35.5 (34.5-36.5)		35.5 (34.5-36.5)		35.2 (33.8-36.7)	35.8 (34.3-37.2)	
Former	41.4 (39.6-43.1)	37.6 (35.9-39.3)		37.6 (35.9-39.3)		41.4 (39.3-43.4)	38.1 (36.2-40.1)	

Current	35.4 (33.1-37.6)	37.6 (35.4-39.9)		37.6 (35.4-39.9)		37.7 (346-40.8)	40.8 (37.7-44.0)	
Binge drinking in past 30 d			0.05		0.05			0.10
Yes	31.7 (29.5-33.8)	34.3 (32.2-36.5)		34.3 (32.2-36.5)		30.7 (28.1-33.4)	34.9 (32.0-37.8)	
No	37.0 (36.2-37.9)	36.6 (35.7-37.5)		36.6 (35.7-37.5)		38.2 (37.0-39.4)	37.4 (36.3-38.6)	
Ever had a skin exam			0.04		0.04			0.08
Yes	40.0 (38.1-41.9)	37.9 (36.1-39.8)		38.0 (36.1-39.9)		40.6 (38.3-42.8)	38.6 (36.5-40.8)	
No	35.1 (34.1-36.1)	35.8 (34.8-36.7)		35.7 (34.8-36.7)		35.5 (34.2-36.9)	36.3 (35.0-37.7)	
Personal history of melanoma			0.06		0.06			0.05
Yes	61.6 (52.1-71.1)	45.6 (35.9-55.7)		45.7 (35.9-55.8)		65.7 (55.7-75.7)	47.1 (37.0-57.4)	
No	36.1 (35.2-36.9)	36.2 (35.3-37.1)		36.2 (35.3-37.1)		36.8 (35.7-37.9)	36.9 (35.8-38.1)	
Family history of melanoma			0.26		0.25			0.37
Yes	34.4 (29.5-39.3)	33.7 (29.5-38.3)		33.7 (29.4-38.2)		34.8 (29.1-40.6)	34.7 (29.7-40.0)	
No	36.2 (35.4-37.2)	36.3 (35.5-37.2)		36.3 (35.5-37.2)		37.1 (36.0-38.2)	37.1 (36.0-38.3)	
Ever had cancer			0.007		0.008			0.003
Yes	47.3 (44.5-50.2)	39.7 (37.1-42.4)		39.7 (37.1-42.4)		51.4 (47.5-55.3)	42.0 (38.5-45.6)	
No	35.3 (34.4-36.1)	35.9 (35.0-36.8)		35.9 (35.0-36.9)		35.5 (34.3-36.7)	36.5 (35.3-37.7)	
Indoor tanning			<0.0001		<0.0001			<0.0001
Yes	14.2 (11.4-17.0)	24.0 (20.4-28.1)		24.1 (20.4-28.2)		13.6 (10.1-17.2)	24.1 (19.4-29.5)	
No	37.1 (36.2-38.0)	36.6 (35.7-37.5)		36.6 (35.7-37.5)		38.1 (36.9-39.3)	37.5 (36.3-38.6)	
Sunless tanning (self-applied)			0.17		0.17			0.20
Yes	26.3 (23.4-29.2)	34.0 (30.7-37.4)		33.9 (30.7-37.3)		27.1 (23.8-30.5)	34.7 (31.2-38.4)	
No	36.8 (35.9-37.7)	36.4 (35.5-37.3)		36.4 (35.5-37.3)		37.9 (36.7-39.1)	37.2 (36.0-38.4)	
Insurance status			0.006		0.005			0.71
Uninsured	40.0 (37.6-42.5)	39.5 (37.0-42.0)		39.6 (37.1-42.1)		40.1 (36.5-43.7)	37.7 (34.0-41.5)	
Insured	35.8 (34.9-36.7)	35.9 (35.0-36.8)		35.9 (35.0-36.8)		36.7 (35.5-37.9)	36.9 (35.7-38.2)	

[a]Sun-sensitivity defined as reporting any skin burn when not protected from the sun for 1 hour.
[b]Sun protective clothing was defined as always or usually wearing at least 1: wide-brimmed hat, long sleeved shirt, or long clothing to the ankles.
[c]Reported walking categories for at least 10 minutes in the past 7 days.
[d]Overall significant contrasts, without leisure-time physical activity covariate: no reported walking vs both, p<0.0001; no reported walking vs transportation, p=0.03; leisure vs both, p=0.0003.
[e]Overall significant contrasts: no reported walking vs both, p<0.0001; no reported walking vs. transportation, p=0.04; leisure vs both, p=0.0002.
[f]Sun-sensitive significant contrasts: no reported walking vs both, p=0.007; leisure vs both, p=0.01.
[g]Regular sunscreen use was defined as always or usually using sunscreen with SPF15+ (sun protection factor).
[h]Sun avoidance was defined as always or usually staying in the shade or responding not going into the sun for any sun protective question.
[i]Individuals were categorized into 4 activity levels based on the 2008 Physical Activity Guidelines for Americans: highly active (>300 min/week of light or moderate-intensity aerobic activity, 150 minutes of vigorous-intensity aerobic activity, or an equivalent combination per week [i.e., moderate-intensity equivalent activity]), sufficiently active (150–300 min/week of moderate-intensity equivalent activity), insufficiently active (some activity but less than 150 min/week of moderate-intensity equivalent activity), and inactive (no light to moderate or vigorous-intensity aerobic activity for at least 10 minutes).

Supplement Table 4. Weighted and adjusted odds ratios for US adults and sun-sensitive individuals[a] aged ≥ 18 years for regular sunscreen use[b] by quartile of weekly transportation and leisure walking minutes[c] and covariates, National Health Interview Survey, 2015

	Overall				Sun-sensitive	
	Adjusted OR (without physical activity level), sunscreen use (95% CI)	*P*	Adjusted OR, sunscreen use (95% CI)	*P*	Adjusted OR, sunscreen use (95% CI)	*P*
Gender		<0.0001		<0.0001		<0.0001
Women	ref		ref		ref	
Men	0.4 (0.4-0.4)		0.4 (0.3-0.4)		0.4 (0.4-0.5)	
Transportation walking (weekly minutes)		0.008		0.02		0.02
No reported walking (<10)	ref		ref		ref	
10-30	1.1 (0.9-1.2)		1.0 (0.9-1.2)		1.0 (0.9-1.2)	
31-60	1.4 (1.2-1.6)		1.3 (1.1-1.6)		1.4 (1.1-1.6)	
61-120	1.1 (1.0-1.3)		1.1 (0.9-1.3)		1.2 (0.9-1.4)	
>120	1.1 (0.9-1.3)		1.0 (0.9-1.2)		1.1 (0.9-1.4)	
Leisure walking (weekly minutes)		<0.0001		<0.0001		0.0005
No reported walking (<10)	ref		ref		ref	
10-40	1.3 (1.1-1.4)		1.2 (1.0-1.4)		1.1 (1.0-1.3)	
41-80	1.4 (1.3-1.6)		1.3 (1.2-1.5)		1.3 (1.2-1.6)	
81-150	1.5 (1.3-1.6)		1.3 (1.1-1.5)		1.3 (1.1-1.5)	
>150	1.6 (1.4-1.8)		1.3 (1.2-1.6)		1.3 (1.1-1.6)	
Needs walking assistance		<0.0001		<0.0001		<0.0001
Yes	0.5 (0.4-0.6)		0.6 (0.5-0.7)		0.6 (0.4-0.7)	
No	ref		ref		ref	
Age group		<0.0001		<0.0001		<0.0001
18-24	1.2 (1.0-1.5)		1.2 (1.0-1.5)		1.2 (0.9-1.6)	
25-34	1.4 (1.2-1.6)		1.4 (1.2-1.6)		1.3 (1.1-1.6)	
35-44	1.9 (1.6-2.2)		1.8 (1.6-2.1)		1.7 (1.5-2.1)	
45-64	1.4 (1.3-1.7)		1.4 (1.3-1.6)		1.4 (1.2-1.6)	
≥65	ref		ref		ref	
Race/Ethnicity		<0.0001		<0.0001		0.0003
White, non-Hispanic	1.5 (1.3-1.7)		1.5 (1.3-1.7)		1.2 (0.9-1.6)	
Black, non-Hispanic	0.3 (0.3-0.4)		0.3 (0.3-0.4)		0.6 (0.4-0.9)	

Hispanic	1.0 (0.9-1.2)		1.0 (0.9-1.2)		1.0 (0.8-1.3)	
Other race	ref		ref		ref	
Education		<0.0001		<0.0001		<0.0001
Less than high school graduate	0.4 (0.3-0.4)		0.4 (0.3-0.5)		0.4 (0.3-0.6)	
High school graduate	0.5 (0.5-0.6)		0.6 (0.5-0.6)		0.6 (0.5-0.6)	
Some college	0.7 (0.6-0.8)		0.7 (0.7-0.8)		0.7 (0.6-0.8)	
College graduate	ref		ref		ref	
Marital status		<0.0001		<0.0001		0.005
Married or living with partner	1.3 (1.1-1.4)		1.3 (1.1-1.4)		1.3 (1.1-1.5)	
Divorced or separated	1.1 (1.0-1.3)		1.2 (1.0-1.3)		1.2 (1.0-1.4)	
Widowed	1.0 (0.8-1.2)		1.0 (0.8-2.1)		1.0 (0.7-1.2)	
Never married	ref		ref		ref	
Foreign born status		0.16		0.25		0.03
United States born	1.1 (1.0-1.3)		1.1 (1.0-1.3)		1.3 (1.0-1.6)	
<10 y in the United States	0.9 (0.7-1.2)		1.0 (0.8-1.2)		0.9 (0.7-1.3)	
≥10 y in the United States	ref		ref		ref	
US Census region		0.001		0.003		0.21
Northeast	1.1 (0.9-1.2)		1.1 (0.9-1.2)		1.1 (1.0-1.3)	
Midwest	0.8 (0.7-0.9)		0.8 (0.7-1.0)		0.9 (0.8-1.1)	
South	1.0 (0.9-1.1)		1.0 (0.9-1.1)		1.0 (0.9-1.2)	
West	ref		ref		ref	
Sunburn in past 12 months		<0.0001		<0.0001		0.08
Yes	1.4 (1.2-1.9)		1.3 (1.2-1.5)		1.1 (1.0-1.2)	
No	ref		ref		ref	
Skin reaction after 2 weeks in sun		<0.0001		<0.0001		<0.0001
Very dark	1.5 (1.2-1.9)		1.4 (1.2-1.8)		0.9 (0.6-1.4)	
Moderate tan	1.7 (1.4-2.0)		1.6 (1.3-1.9)		0.9 (0.6-1.4)	
Mild tan	1.9 (1.5-2.3)		1.8 (1.5-2.2)		1.1 (0.8-1.7)	
Burn repeatedly or freckle	3.4 (2.8-4.2)		3.3 (2.7-4.1)		1.9 (1.3-2.8)	
Do not go out in the sun	ref		ref		ref	
Regular protective clothing use[d]		<0.0001		<0.0001		0.16
Yes	ref		ref		ref	

No	0.8 (0.7-0.9)		0.8 (0.7-0.9)		0.9 (0.8-1.0)	
Regular sun avoidance[e]		<0.0001		<0.0001		<0.0001
Yes	ref		ref		ref	
No	0.6 (0.6-0.7)		0.6 (0.6-0.7)		0.7 (0.6-0.7)	
Physical activity level[f]		-		<0.0001		<0.0001
Inactive	-		0.6 (0.6-0.7)		0.7 (0.6-0.8)	
Insufficiently active	-		0.7 (0.7-0.8)		0.7 (0.6-0.9)	
Sufficiently active	-		0.9 (0.8-1.0)		1.0 (0.9-1.1)	
Highly active	-		ref		ref	
Obesity or overweight		0.001		0.005		0.03
Yes	ref		ref		ref	
No	1.2 (1.1-1.3)		1.1 (1.0-1.3)		1.1 (1.0-1.3)	
Smoking status		<0.0001		<0.0001		<0.0001
Never	1.9 (1.7-2.2)		1.9 (1.6-2.1)		1.9 (1.6-2.2)	
Former	1.7 (1.5-2.0)		1.6 (1.4-1.9)		1.7 (1.4-2.0)	
Current	ref		ref		ref	
Binge drinking in past 30 d		0.01		0.03		0.08
Yes	ref		ref		ref	
No	0.8 (0.7-1.0)		0.9 (0.8-1.0)		0.9 (0.8-1.0)	
Ever had a skin exam		<0.0001		<0.0001		<0.0001
Yes	1.9 (1.7-2.1)		1.8 (1.7-2.0)		1.8 (1.6-2.0)	
No	ref		ref		ref	
Personal history of melanoma		0.05		0.05		0.74
Yes	1.6 (1.0-2.6)		1.6 (1.0-2.6)		1.1 (0.7-1.8)	
No	ref		ref		ref	
Family history of melanoma		0.26		0.32		0.84
Yes	1.2 (0.9-1.5)		1.1 (0.9-1.4)		1.0 (0.8-1.3)	
No	ref		ref		ref	
Ever had cancer		0.09		0.09		0.10
Yes	1.1 (1.0-1.3)		1.1 (1.0-1.3)		1.2 (1.0-1.4)	
No	ref		ref		ref	
Indoor tanning		0.008		0.006		0.03
Yes	0.8 (0.6-0.9)		0.7 (0.6-0.9)		0.8 (0.6-1.0)	
No	ref		ref		ref	
Sunless tanning (self-applied)		0.04		0.07		0.17
Yes	ref		ref		ref	

No	0.8 (0.7-0.9)		0.9 (0.7-1.0)		0.9 (0.7-1.1)	
Insurance status		0.01		0.01		0.05
Uninsured	0.8 (0.7-1.0)		0.8 (0.7-1.0)		0.8 (0.7-1.0)	
Insured	ref		ref		ref	

OR=odds ratio; ref=reference category

[a]Sun-sensitivity defined as reporting any skin burn when not protected from the sun for 1 hour.

[b]Sunscreen use was defined as always or usually using sunscreen with SPF15+ (sun protection factor).

[c]Reported walking categories for at least 10 minutes in the past 7 days.

[d]Sun protective clothing was defined as always or usually wearing at least 1: wide-brimmed hat, long sleeved shirt, or long clothing to the ankles.

[e]Sun avoidance was defined as always or usually staying in the shade or responding not going into the sun for any sun protective question.

[f]Individuals were categorized into 4 activity levels based on the 2008 Physical Activity Guidelines for Americans: highly active (>300 min/week of light or moderate-intensity aerobic activity, 150 minutes of vigorous-intensity aerobic activity, or an equivalent combination per week [i.e., moderate-intensity equivalent activity]), sufficiently active (150–300 min/week of moderate-intensity equivalent activity), insufficiently active (some activity but less than 150 min/week of moderate-intensity equivalent activity), and inactive (no light to moderate or vigorous-intensity aerobic activity for at least 10 minutes).

Supplement Table 5. Weighted and adjusted odds ratios for US adults and sun-sensitive individuals[a] aged ≥ 18 years for regular sun avoidance[b] by quartile of weekly transportation and leisure walking minutes[c] and covariates, National Health Interview Survey, 2015

	Overall				Sun-sensitive	
	Adjusted OR (without physical activity level), sun avoidance (95% CI)	*P*	Adjusted OR, sun avoidance (95% CI)	*P*	Adjusted OR, sun avoidance (95% CI)	*P*
Gender		<0.0001		<0.0001		<0.0001
Women	ref		ref		ref	
Men	0.6 (.5-.6)		0.6 (0.5-0.6)		0.6 (0.5-0.7)	
Transportation walking (weekly minutes)		0.02		0.05		0.35
No reported walking (<10)	ref		ref		ref	
10-30	1.1 (0.9-1.2)		1.1 (0.9-1.2)		1.1 (0.9-1.3)	
31-60	1.0 (0.9-1.2)		1.0 (0.9-1.2)		1.0 (0.8-1.2)	
61-120	0.8 (0.7-1.0)		0.8 (0.7-1.0)		0.9 (0.7-1.1)	
>120	0.8 (0.7-1.0)		0.9 (0.8-1.0)		1.1 (0.8-1.3)	
Leisure walking (weekly minutes)		<0.0001		0.0008		0.009
No reported walking (<10)	ref		ref		ref	
10-40	1.0 (0.9-1.1)		1.0 (0.9-1.2)		1.0 (0.9-1.2)	
41-80	0.8 (0.7-0.9)		0.9 (0.8-1.0)		0.9 (0.7-1.0)	
81-150	0.8 (0.7-0.9)		0.8 (0.7-1.0)		0.8 (0.7-0.9)	
>150	0.8 (0.7-0.9)		0.9 (0.8-1.0)		0.8 (0.7-0.9)	
Needs walking assistance		<0.0001		<0.0001		<0.0001
Yes	1.9 (1.6-2.2)		1.8 (1.6-2.1)		2.0 (1.6-2.4)	
No	ref		ref		ref	
Age group		0.01		0.05		0.11
18-24	0.7 (0.6-0.9)		0.7 (0.6-0.9)		0.7 (0.5-0.9)	
25-34	0.8 (0.7-1.0)		0.9 (0.7-1.0)		0.9 (0.7-1.1)	
35-44	0.9 (0.7-1.0)		0.9 (0.8-1.0)		0.9 (0.8-1.2)	
45-64	0.9 (0.8-1.1)		0.9 (0.8-1.1)		0.9 (0.8-1.1)	
≥65	ref		ref		ref	
Race/Ethnicity		<0.0001		<0.0001		<0.0001
White, non-Hispanic	0.7 (0.6-0.8)		0.7 (0.6-0.8)		0.8 (0.6-1.0)	
Black, non-Hispanic	1.4 (1.2-1.7)		1.4 (1.2-1.7)		1.6 (1.1-2.2)	

Hispanic	1.2 (1.0-1.4)		1.2 (1.0-1.4)		1.2 (0.9-1.6)	
Other race	ref		ref		ref	
Education		0.02		0.04		0.12
Less than high school graduate	1.1 (0.9-1.2)		1.0 (0.9-1.2)		1.1 (0.9-1.3)	
High school graduate	1.1 (1.0-1.2)		1.1 (1.0-1.2)		1.2 (1.0-1.4)	
Some college	1.2 (1.1-1.3)		1.2 (1.0-1.3)		1.1 (1.0-1.3)	
College graduate	ref		ref		ref	
Marital status		0.0001		0.0001		0.005
Married or living with partner	0.9 (0.8-1.0)		0.9 (0.8-1.0)		0.8 (0.7-1.0)	
Divorced or separated	0.8 (0.7-0.9)		0.8 (0.7-0.9)		0.8 (0.6-0.9)	
Widowed	1.1 (0.9-1.3)		1.1 (0.9-1.3)		1.0 (0.8-1.4)	
Never married	ref		ref		ref	
Foreign born status		0.003		0.004		0.0001
United States born	0.8 (0.7-0.9)		0.8 (0.7-0.9)		0.7 (0.6-0.8)	
<10 y in the United States	1.0 (0.8-1.2)		1.0 (0.8-1.2)		0.9 (0.6-1.3)	
≥10 y in the United States	ref		ref		ref	
US Census region		<0.0001		<0.0001		<0.0001
Northeast	0.9 (0.7-1.0)		0.8 (0.7-1.0)		0.9 (0.7-1.0)	
Midwest	0.6 (0.6-0.7)		0.6 (0.5-0.7)		0.6 (0.5-0.7)	
South	1.0 (0.9-1.1)		1.0 (0.9-1.1)		1.0 (0.9-1.1)	
West	ref		ref		ref	
Sunburn in past 12 months		<0.0001		<0.0001		<0.0001
Yes	0.8 (0.7-0.9)		0.8 (0.7-0.9)		0.7 (0.6-0.8)	
No	ref		ref		ref	
Skin reaction after 2 weeks in sun		<0.0001		<0.0001		<0.0001
Very dark	0.1 (0.08-0.1)		0.1 (0.08-0.1)		0.3 (0.2-0.4)	
Moderate tan	0.1 (0.08-0.1)		0.1 (0.08-0.1)		0.2 (0.2-0.3)	
Mild tan	0.1 (0.08-0.1)		0.1 (0.08-0.1)		0.2 (0.2-0.3)	
Burn repeatedly or freckle	0.2 (0.2-0.3)		0.2 (0.08-0.1)		0.5 (0.3-0.7)	
Do not go out in the sun	ref		ref		ref	
Regular protective clothing use[d]		<0.0001		<0.0001		<0.0001
Yes	ref		ref		ref	

No	0.5 (0.4-0.5)		0.5 (0.4-0.5)		0.4 (0.4-0.5)	
Regular sunscreen use[e]		<0.0001		<0.0001		<0.0001
Yes	ref		ref		ref	
No	0.6 (0.6-0.7)		0.6 (0.6-0.7)		0.7 (0.6-0.8)	
Physical activity level[f]		-		<0.0001		<0.0001
Inactive	-		1.4 (1.2-1.5)		1.4 (1.2-1.7)	
Insufficiently active	-		1.4 (1.3-1.6)		1.5 (1.3-1.7)	
Sufficiently active	-		1.2 (1.1-1.4)		1.2 (1.1-1.4)	
Highly active	-		ref		ref	
Obesity or overweight		<0.0001		<0.0001		<0.0001
Yes	ref		ref		ref	
No	0.8 (0.7-0.8)		0.8 (0.7-0.8)		0.7 (0.6-0.8)	
Smoking status		0.94		0.86		0.58
Never	1.0 (0.9-1.1)		1.0 (0.9-1.2)		1.1 (0.9-1.2)	
Former	1.0 (0.9-1.1)		1.0 (0.9-1.1)		1.0 (0.8-1.1)	
Current	ref		ref		ref	
Binge drinking in past 30 d		0.005		0.01		0.001
Yes	ref		ref		ref	
No	1.2)1.1-1.3)		1.2 (1.0-1.3)		1.3 (1.1-1.5)	
Ever had a skin exam		0.009		0.004		0.03
Yes	1.1 (1.0-1.2)		1.1 (1.0-1.3)		1.1 (1.0-1.3)	
No	ref		ref		ref	
Personal history of melanoma		0.12		0.12		0.08
Yes	1.5 (0.9-2.4)		1.5 (0.9-2.4)		1.6 (0.9-2.8)	
No	ref		ref		ref	
Family history of melanoma		0.91		0.82		0.98
Yes	1.0 (0.8-1.3)		1.0 (0.8-1.3)		1.0 (0.8-1.3)	
No	ref		ref		ref	
Ever had cancer		0.82		0.85		0.96
Yes	1.0 (0.9-1.2)		1.0 (0.9-1.2)		1.0 (0.8-1.2)	
No	ref		ref		ref	
Indoor tanning		<0.0001		<0.0001		<0.0001
Yes	0.5 (0.4-0.7)		0.5 (0.4-0.7)		0.5 (0.4-0.7)	
No	ref		ref		ref	
Sunless tanning (self-applied)		0.28		0.33		0.95
Yes	ref		ref		ref	

No	1.1 (0.9-1.3)		1.1 (0.9-1.3)		1.0 (0.8-1.2)	
Insurance status		0.86		0.85		0.19
Uninsured	1.0 (0.9-1.2)		1.0 (0.9-1.2)		1.1 (0.9-1.2)	
Insured	ref		ref		ref	

OR=odds ratio; ref=reference category

[a]Sun-sensitivity defined as reporting any skin burn when not protected from the sun for 1 hour.

[b]Sun avoidance was defined as always or usually staying in the shade or responding not going into the sun for any sun protective question.

[c]Reported walking categories for at least 10 minutes in the past 7 days.

[d]Sun protective clothing was defined as always or usually wearing at least 1: wide-brimmed hat, long sleeved shirt, or long clothing to the ankles.

[e]Sunscreen use was defined as always or usually using sunscreen with SPF15+ (sun protection factor).

[f]Individuals were categorized into 4 activity levels based on the 2008 Physical Activity Guidelines for Americans: highly active (>300 min/week of light or moderate-intensity aerobic activity, 150 minutes of vigorous-intensity aerobic activity, or an equivalent combination per week [i.e., moderate-intensity equivalent activity]), sufficiently active (150–300 min/week of moderate-intensity equivalent activity), insufficiently active (some activity but less than 150 min/week of moderate-intensity equivalent activity), and inactive (no light to moderate or vigorous-intensity aerobic activity for at least 10 minutes).

Supplement Table 6. Weighted and adjusted odds ratios for US adults and sun-sensitive individuals[a] aged ≥ 18 years for regular sun protective clothing use[b] by quartile of weekly transportation and leisure walking minutes[c] and covariates, National Health Interview Survey, 2015

			Overall		Sun-sensitive	
	Adjusted OR (without physical activity level), protective clothing use (95% CI)	*P*	Adjusted OR, protective clothing use (95% CI)	*P*	Adjusted OR, protective clothing use (95% CI)	*P*
Gender		<0.0001		<0.0001		<0.0001
Women	ref		ref		ref	
Men	1.9 (1.8-2.0)		1.9 (1.8-2.1)		1.8 (1.6-2.0)	
Transportation walking (weekly minutes)		0.0002		0.0002		0.06
No reported walking (<10)	ref		ref		ref	
10-30	1.1 (1.0-1.3)		1.1 (1.0-1.3)		1.0 (0.9-1.2)	
31-60	1.2 (1.1-1.4)		1.2 (1.1-1.4)		1.2 (1.0-1.5)	
61-120	1.1 (1.0-1.3)		1.1 (1.0-1.3)		1.2 (0.9-1.4)	
>120	1.3 (1.2-1.5)		1.3 (1.2-1.6)		1.3 (1.1-1.6)	
Leisure walking (weekly minutes)		0.12		0.17		0.11
No reported walking (<10)	ref		ref		ref	
10-40	1.1 (1.0-1.2)		1.1 (0.9-1.2)		1.0 (0.8-1.2)	
41-80	1.1 (1.0-1.2)		1.1 (0.9-1.2)		1.0 (0.9-1.2)	
81-150	1.1 (1.0-1.3)		1.1 (1.0-1.3)		1.2 (1.0-1.3)	
>150	1.1 (1.0-1.2)		1.1 (1.0-1.3)		1.2 (1.0-1.4)	
Needs walking assistance		0.58		0.53		0.63
Yes	1.0 (0.9-1.2)		1.0 (0.9-1.2)		1.1 (0.9-1.3)	
No	ref		ref		ref	
Age group		<0.0001		<0.0001		<0.0001
18-24	0.4 (0.3-0.4)		0.4 (0.3-0.4)		0.4 (0.3-0.5)	
25-34	0.4 (0.4-0.5)		0.4 (0.4-0.5)		0.4 (0.4-0.5)	
35-44	0.5 (0.4-0.5)		0.5 (0.4-0.5)		0.5 (0.4-0.6)	
45-64	0.7 (0.6-0.7)		0.7 (0.6-0.7)		0.6 (0.5-0.7)	
≥65	ref		ref		ref	
Race/Ethnicity		0.0001		0.0001		0.001
White, non-Hispanic	0.8 (0.7-1.0)		0.8 (0.7-1.0)		0.9 (0.7-1.1)	
Black, non-Hispanic	1.0 (0.9-1.2)		1.0 (0.9-1.2)		1.2 (0.8-1.7)	

Hispanic	1.1 (0.9-1.2)		1.1 (0.9-1.2)		1.2 (0.9-1.6)	
Other race	ref		ref		ref	
Education		<0.0001		<0.0001		0.03
Less than high school graduate	1.4 (1.2-1.6)		1.4 (1.2-1.6)		1.3 (1.1-1.6)	
High school graduate	1.2 (1.1-1.4)		1.2 (1.1-1.4)		1.2 (1.0-1.3)	
Some college	1.1 (1.0-1.2)		1.1 (1.0-1.2)		1.0 (0.9-1.2)	
College graduate	ref		ref		ref	
Marital status		0.32		0.33		0.02
Married or living with partner	1.0 (0.9-1.1)		1.0 (0.9-1.1)		0.8 (0.7-1.0)	
Divorced or separated	0.9 (0.8-1.1)		0.9 (0.8-1.1)		0.8 (0.6-0.9)	
Widowed	1.1 (0.9-1.3)		1.1 (0.9-1.3)		1.0 (0.8-1.3)	
Never married	ref		ref		ref	
Foreign born status		<0.0001		<0.0001		0.004
United States born	0.8 (0.7-0.9)		0.8 (0.7-0.9)		0.8 (0.6-0.9)	
<10 y in the United States	1.3 (1.0-1.5)		1.3 (1.0-1.5)		1.2 (0.8-1.7)	
≥10 y in the United States	ref		ref		ref	
US Census region		<0.0001		<0.0001		<0.0001
Northeast	0.5 (0.4-0.5)		0.5 (0.4-0.5)		0.5 (0.4-0.6)	
Midwest	0.7 (0.6-0.8)		0.7 (0.6-0.8)		0.7 (0.6-0.8)	
South	0.7 (0.6-0.8)		0.7 (0.6-0.8)		0.7 (0.6-0.8)	
West	ref		ref		ref	
Sunburn in past 12 months		0.42		0.41		0.90
Yes	1.0 (0.9-1.1)		1.0 (0.9-1.1)		1.0 (0.9-1.1)	
No	ref		ref		ref	
Skin reaction after 2 weeks in sun		<0.0001		<0.0001		0.0004
Very dark	2.4 (1.9-2.9)		2.3 (1.9-2.8)		1.0 (0.7-1.5)	
Moderate tan	2.2 (1.9-2.6)		2.2 (1.8-2.6)		1.0 (0.7-1.5)	
Mild tan	2.4 (2.0-2.9)		2.4 (2.0-2.8)		1.1 (0.8-1.5)	
Burn repeatedly or freckle	3.0 (2.5-3.6)		2.9 (2.5-3.5)		1.4 (1.0-1.9)	
Do not go out in the sun	ref		ref		ref	
Regular sun avoidance[d]		<0.0001		<0.0001		<0.0001
Yes	ref		ref		ref	
No	0.5 (0.4-0.5)		0.5 (0.4-0.5)		0.4 (0.4-1.4)	

Regular sunscreen use[e]		<0.0001		<0.0001		0.14
Yes	ref		ref		ref	
No	0.8 (0.7-0.9)		0.8 (0.7-0.9)		0.9 (0.8-1.0)	
Physical activity level[f]		-		0.10		0.42
Inactive	-		1.0 (0.9-1.1)		1.1 (0.9-1.2)	
Insufficiently active	-		1.1 (1.0-1.3)		1.1 (1.0-1.3)	
Sufficiently active	-		1.0 (0.9-1.1)		1.0 (0.9-1.1)	
Highly active	-		ref		ref	
Obesity or overweight		0.26		0.25		0.33
Yes	ref		ref		ref	
No	1.0 (1.0-1.1)		1.1 (1.0-1.1)		1.1 (0.9-1.2)	
Smoking status		0.03		0.04		0.007
Never	0.9 (0.8-1.0)		0.9 (0.8-1.0)		0.8 (0.7-0.9)	
Former	1.0 (0.9-1.1)		1.0 (0.9-1.1)		0.9 (0.7-1.0)	
Current	ref		ref		ref	
Binge drinking in past 30 d		0.05		0.05		0.11
Yes	ref		ref		ref	
No	1.1 (1.0-1.2)		1.1 (1.0-1.2)		1.1 (1.0-1.3)	
Ever had a skin exam		0.06		0.06		0.10
Yes	1.1 (1.0-1.2)		1.1 (1.0-1.2)		1.1 (1.0-1.3)	
No	ref		ref		ref	
Personal history of melanoma		0.06		0.06		0.05
Yes	1.6 (1.0-2.5)		1.6 (1.0-2.5)		1.6 (1.0-2.6)	
No	ref		ref		ref	
Family history of melanoma		0.28		0.27		0.38
Yes	0.9 (0.7-1.1)		0.9 (0.7-1.1)		0.9 (0.7-1.2)	
No	ref		ref		ref	
Ever had cancer		0.007		0.007		0.003
Yes	1.2 (1.1-1.4)		1.2 (1.1-1.2)		1.3 (1.1-1.5)	
No	ref		ref		ref	
Indoor tanning		<0.0001		<0.0001		<0.0001
Yes	0.5 (0.4-0.6)		0.5 (0.4-0.6)		0.5 (0.4-0.7)	
No	ref		ref		ref	
Sunless tanning (self-applied)		0.19		0.19		0.26
Yes	ref		ref		ref	
No	1.1 (0.9-1.3)		1.1 (0.9-1.3)		1.1 (0.9-1.4)	

Insurance status		0.005		0.004		0.71
Uninsured	1.2 (1.1-1.4)		1.2 (1.1-1.4)		1.0 (0.9-1.3)	
Insured	ref		ref		ref	

OR=odds ratio; ref=reference category

[a]Sun-sensitivity defined as reporting any skin burn when not protected from the sun for 1 hour.

[b]Sun protective clothing was defined as always or usually wearing at least 1: wide-brimmed hat, long sleeved shirt, or long clothing to the ankles.

[c]Reported walking categories for at least 10 minutes in the past 7 days.

[d]Sun avoidance was defined as always or usually staying in the shade or responding not going into the sun for any sun protective question.

[e]Sunscreen use was defined as always or usually using sunscreen with SPF15+ (sun protection factor)

[f] Individuals were categorized into 4 activity levels based on the 2008 Physical Activity Guidelines for Americans: highly active (>300 min/week of light or moderate-intensity aerobic activity, 150 minutes of vigorous-intensity aerobic activity, or an equivalent combination per week [i.e., moderate-intensity equivalent activity]), sufficiently active (150–300 min/week of moderate-intensity equivalent activity), insufficiently active (some activity but less than 150 min/week of moderate-intensity equivalent activity), and inactive (no light to moderate or vigorous-intensity aerobic activity for at least 10 minutes)
